# Targeting ZFP64/GAL-1 axis promotes therapeutic effect of nab-paclitaxel and reverses immunosuppressive microenvironment in gastric cancer

**DOI:** 10.1186/s13046-021-02224-x

**Published:** 2022-01-07

**Authors:** Mengxuan Zhu, Pengfei Zhang, Shan Yu, Cheng Tang, Yan Wang, Zhenbin Shen, Weidong Chen, Tianshu Liu, Yuehong Cui

**Affiliations:** 1 Department of Medical Oncology, Zhongshan Hospital, Fudan University, Shanghai, 200032 China; 2 Center of Evidence-based Medicine, Fudan University, Shanghai, China; 3 Department of General Surgery, Zhongshan Hospital, Fudan University, Shanghai, China

**Keywords:** Gastric cancer, Chemoresistance, ZFP64, Cancer stem cell, Immunosuppression

## Abstract

**Background:**

Chemoresistance is a main obstacle in gastric cancer (GC) treatment, but its molecular mechanism still needs to be elucidated. Here, we aim to reveal the underlying mechanisms of nanoparticle albumin-bound paclitaxel (nab-paclitaxel) resistance in GC.

**Methods:**

We performed RNA sequencing (RNA-seq) on samples from patients who were resistant or sensitive to nab-paclitaxel, and identified Zinc Finger Protein 64 (ZFP64) as critical for nab-paclitaxel resistance in GC. CCK8, flow cytometry, TUNEL staining, sphere formation assays were performed to investigate the effects of ZFP64 in vitro, while subcutaneous tumor formation models were established in nude mice or humanized mice to evaluate the biological roles of ZFP64 in vivo. Chromatin immunoprecipitation sequencing (CHIP-seq) and double-luciferase reporter gene assay were conducted to reveal the underlying mechanism of ZFP64.

**Results:**

ZFP64 overexpression was linked with aggressive phenotypes, nab-paclitaxel resistance and served as an independent prognostic factor in GC. As a transcription factor, ZFP64 directly binds to Galectin-1 (GAL-1) promoter and promoted GAL-1 transcription, thus inducing stem-cell like phenotypes and immunosuppressive microenvironment in GC. Importantly, compared to treatment with nab-paclitaxel alone, nab-paclitaxel plus GAL-1 blockade significantly enhanced the anti-tumor effect in mouse models, particularly in humanized mice.

**Conclusions:**

Our data support a pivotal role for ZFP64 in GC progression by simultaneously promoting cellular chemotherapy resistance and tumor immunosuppression. Treatment with the combination of nab*-*paclitaxel and a GAL-1 inhibitor might benefit a subgroup of GC patients.

**Supplementary Information:**

The online version contains supplementary material available at 10.1186/s13046-021-02224-x.

## Background

Gastric cancer is the 2nd most common malignancy worldwide and the third main cause of global cancer-related mortality [1]. Surgery remains the only potentially curative approach for this disease, but the majority of patients with GC are diagnosed at unresectable stages due to a lack of early symptoms and effective screening methods, and chemotherapy is the preferred option for these patients [2]. Although numerous novel chemotherapeutic medications, such as nab-paclitaxel have been introduced into clinical practice, chemotherapeutic approaches fail frequently due to intrinsic or acquired drug resistance [3]. Resistance to chemotherapy occurs intrinsically before the administration of chemotherapy or during chemotherapy, and mechanisms of chemotherapy resistance in GC are complex and multifactorial, including reduced intracellular concentrations of drugs, alterations in drug targets, dysregulation of cell survival and death pathways [4–6]. Currently, the underlaying mechanism of nab-paclitaxel resistance in GC remains ambiguous and needs a deeper understanding at the molecular level to benefit a subset of patients with GC.

Among the confirmed mechanisms of chemoresistance, cancer stem cells (CSCs) has recently been characterized as being responsible for resistance to chemotherapy. Indeed, an infrequent subset of CSCs among tumor masses was observed in GC patient-derived subcutaneous xenografts (PDX) in nude mice, which contributed to cancer initiation and chemoresistance. Emerging evidence has revealed the vital contribution of CSCs to intratumor heterogeneity, which further enables tumor cells to survive upon exposure to chemotherapy. As the earliest marker of CSCs in GC, CD44 was found to be positively associated with the resistance of GC cells, and inhibition of CD44 might represent a target in the treatment of GC [7]. Importantly, accumulating experimental evidence has revealed that targeting markers of CSCs, related pathways or microenvironmental niches might impair chemoresistance [8, 9]. Despite these debates about the CSC origin, stemness maintenance and continuous differentiation, an urgent and continuous need is to improve our understanding of the function and behavior of CSCs in cancer progression and chemoresistance.

Zinc Finger Protein 64 (ZFP64, also named ZNF338), a member of the Krüppel C2H2-type zinc finger family, is a putative transcriptional regulator [10]. To date, the biological function of ZFP64 largely remains to be defined, and limited data have indicated that ZFP64 mediated mesenchymal cell differentiation by modulating Notch signaling [11]. A recent study identified a critical role for ZFP64 as a TF (transcriptional factor) with an important role in maintaining the expression of the *MLL* oncogene in MLL (mixed lineage leukemia gene-rearranged leukemia) [12]. Thus, ZFP64 may play a vital role in tumor development. Indeed, several high-throughput genomic and proteomic analyses revealed that ZFP64 was upregulated in tumor tissues compared to the corresponding paracancerous tissues and might be a novel potential oncogene [13, 14]. Although few studies on ZFP64 have been conducted, ZFP64 has been proposed to be involved in the immune response and tumorigenesis. Based on the dysregulation of several oncogenic and tumor suppressor genes that directly regulate immune checkpoint expression and result in immunosuppression, ZFP64 might also be a bona fide driver of GC and a direct regulator of the tumor immune microenvironment in GC.

Here, we revealed increased ZFP64 expression in GC compared to adjacent nontumor specimens, and upregulation of ZFP64 was more obvious in GC tissues from patients who were resistant to nab-paclitaxel. Moreover, high ZFP64 expression was associated with a poor prognosis of patients with GC, which was further supported by the in vitro study. Based on the ChIP-seq and promoter luciferase activity analyses, ZFP64 activated Gal-1 transcription by binding to the Gal-1 promoter, which induced cellular acquisition of the CSC phenotype and established an immunosuppressive tumor microenvironment. Thus, we identified a pivotal role for ZFP64 in GC progression by simultaneously promoting cellular drug resistance and tumor immunosuppression and revealed a novel mechanism of GC chemoresistance. Treatment with GAL-1 inhibitors increased the sensitivity of GC to nab-paclitaxel, which deserves further investigation in clinical practice.

## Methods

### Clinical data from patients and follow-up

First, we prospectively followed some patients who received neoadjuvant chemotherapy, and the chemotherapy regimen was the AS regimen (nab-paclitaxel 125 mg/m^2^ on d1 and d8, and S-1 80 mg/m^2^ on d1–14, q21d). According to the Becker Criteria of Germany [15], the patients were divided into two groups: patients who were sensitive to neoadjuvant chemotherapy: residual tumors were less than 50% of the primary lesion; and patients who were resistant to neoadjuvant chemotherapy: progression after neoadjuvant chemotherapy. Then, the tumor and paired paracancerous samples from 4 patients with GC (2 patients resistant and 2 patients sensitive to neoadjuvant chemotherapy) were analyzed using next-generation sequencing (NGS) [16]. Four hundred twenty tumor and paired paracancerous specimens were randomly collected from patients who underwent curative resection of GC between January 2004 and August 2008 at Zhongshan Hospital, Fudan University (Shanghai, China), and further used for tissue microarray (TMA) construction. The diagnosis of GC was independently confirmed by two pathologists. The study was approved by the Institutional Review Board of Zhongshan Hospital Fudan University, and informed consent was obtained from all patients prior to study.

### Cell lines and transfection

All cell lines were purchased from the Chinese Academy of Science Cell Bank (Shanghai, China). Cells were cultured in RPMI-1640 medium supplemented with 10% fetal bovine serum and 1% antibiotics at 37 °C in a humidified incubator with 5% CO_2_.

ZFP64 short hairpin RNA (shRNA) lentivirus (ZFP64 shRNA), GAL-1 short hairpin RNA (shRNA) lentivirus (GAL-1 shRNA), and ZFP64–overexpressing lentivirus were synthesized by Genomeditech (Shanghai). The shRNA targeting sequences were as follows: ZFP64 shRNA#1: 5′-CTATCCAGGTTGCCAGTTCA-3′, ZFP64 shRNA#2: 5′-TTCTGGTGGTGGTCTGAGTC-3′. GAL-1 shRNA: 5′-CCGGCCTACACTTCAATCCTCGC TTCTCGAGAAGCGAGGATTGAAGTGTAGGTTTTT-3′.

### Immunohistochemistry (IHC) and western blot analysis

The IHC process and the quantification of staining intensity were performed as described in previous studies [17, 18]. Western blot analysis was performed using the method described in our previous study [19]. Relative protein expression was analyzed using Image-Pro Plus software and ImageJ software. The primary antibodies are listed in Table S1.

### RNA isolation and quantitative real-time PCR analysis

For qRT-PCR, total RNA was extracted from GC samples and cells as previously described [17], and amplified with SYBR Green Real-time PCR Master Mix (Yeasen, Shanghai, China). The PCR primers are listed in Table S2.

### Cell migration and invasion assays

Cell migration and Matrigel invasion assays were performed as described in our previous report [17]. For the wound healing assay, cells were seeded in six-well plates and scratched with sterile 200 μl pipette tips to artificially create wounds. The wound healing process was observed and photographed at the indicated time points. For the Matrigel invasion assay, cells were added to 250 μl of serum-free media and seeded in the upper chamber of the transwell insert. The upper chamber was then transferred to a well containing 500 μl of media supplemented with 10% FBS and incubated for 18 h. Cells may actively migrate from the upper to the lower side of the filter using FBS as an attractant. Cells on the upper side were removed using cotton swabs, and the invasive cells on the lower side were fixed, stained with a 0.2% crystal violet solution, and counted under a light microscope. The experiment was repeated three times.

### Chemoresistance assay

Chemotherapy-induced cytotoxicity was assayed using the Cell Counting Kit-8 assay (Yeasen, Shanghai, China). Briefly, cells were plated in 96-well plates, allowed to attach overnight, and then treated with nab-paclitaxel for different time points (AGS, 15 nM; MGC-803, 5 nM; HGC-27, 5 nM). The Cell Counting Kit-8 assay was performed according to the manufacturer’s protocols.

### Colony formation assays

GC cells were seeded in 6-well plates at a density of 1000 cells/well. The colonies propagated in culture and were visible (more than 50 cells) at day 14. Then, the colonies were fixed with 4% paraformaldehyde and stained with Giemsa (Yeasen, Shanghai, China), and the number of colonies in each well was determined under an optical microscope.

### Sphere formation assay

The sphere formation assay was carried out as described in a previous study [20]. Briefly, 100 cancer cells were plated in an ultralow attachment 96-well plate (Corning Inc., USA) in 100 μl of FBS-free RIPM-1640 medium supplemented with L-glutamine (2 mM), 20 ng/ml human EGF (epidermal growth factor), 20 ng/ml human FGF2 (fibroblast growth factor-2), and B-27 supplement (1:50) at 37 °C for 2 weeks. Subsequently, the diameters of the spheres were measured, and the number of spheres with a diameter > 100 μm were considered primary spheres.

### Enzyme-linked immunosorbent assay (ELISA)

The GAL-1 concentration in the serum from the peripheral blood of patients with GC and in the supernatants of different GC cell lines was measured using an ELISA (RayBiotech, Norcross, GA) according to the manufacturer’s instructions.

### Flow cytometry assays and CyTOF staining

For flow cytometry, cells were stained with Annexin V-FITC, CD8-PE and CD44-APC antibodies. Stained samples were analyzed using the BD FACSAria III cytometer. The CyTOF Staining was performed as previous report [21].

### TUNEL staining assay

Paraffin-embedded tissue sections were stained with the TUNEL Apoptosis Detection Kit (Alexa Fluor 640) (Yeasen, Shanghai, China) according to the manufacturer’s protocol. Images were acquired with an Olympus fluorescence microscope (FV1000; Olympus, Tokyo, Japan).

### Chromatin immunoprecipitation assay (ChIP assay)

The ChIP assay was performed as described previously [22]. Briefly, 3 × 10^6^ AGS-ZFP64 cells were harvested and cross-linked with 1% formaldehyde for 10 min at 37 °C. Cross-links were terminated with 1 M glycine, washed with PBS, and cells were resuspended in RIPA buffer. Lysates were sonicated and centrifuged for 15–20 min, and the pellets (including chromatin fragments 200–800 bp) were suspended and diluted 1:10 in nuclear lysis buffer. The chromatin was precleared with a 50% salmon sperm DNA/Protein A agarose slurry (EMD Millipore) and then incubated with the ZFP64 (5 μg) antibody at 4 °C overnight on a rotating platform. The quality of the DNA sample was verified using RT-PCR. Samples were prepared according to the Illumina library protocol and sequenced using the Illumina sequencing system. The quantity of ChIP DNA was determined by normalizing the ratio of input to the.

first amplicon of every gene.

### Dual luciferase reporter assay

The dual luciferase reporter assay was performed as described in a previous report [8]. HEK293 and GC cells were employed in the dual luciferase reporter assay, and all luciferase reporter vectors were constructed by Shanghai Genomeditech Company (Shanghai, China).

### Coculture assay

A coculture assay with HGC-27 cells and T cells was performed as described below. CD3^+^ T cells were isolated from PBMCs using a magnetic selection kit (Stemcell Technologies, Vancouver, Canada) and then stimulated with 25 μl/ml human CD3/CD28 T cell activator (Stemcell Technologies, Vancouver, Canada) and 10 ng/ml human IL-2 (Peprotech, Rocky Hill, NJ). For the contact coculture assay, cancer cells were seeded in 24-well plates. After 24 h, the media were replaced with fresh complete RPMI without cytokines, and T cells were added to the cancer cells at a 1:1 ratio. A CD8 antibody and Annexin V Apoptosis Detection Kit I (BD) were used to detect apoptotic T cells using a FACS flow cytometer at the indicated time points. After 24 h of coculture, the supernatants were collected from cocultures and analyzed using the Human XL Cytokine Array Kit (R&D Systems).

### Human CD34^+^ cell isolation and establishment of humanized mice

Cord blood samples were obtained from pregnant women giving birth to newborns after written consent was obtained from the donors in accordance with the ethical guidelines of Obstetrics and Gynecology Hospital of Fudan University and Zhongshan Hospital of Fudan University, China. Human CD34^+^ cells were isolated and purified using a CD34 MultiSort Kit (Miltenyibiotec) under sterile conditions, according to the manufacturer’s instructions. The purity of the CD34^+^ cells was identified using flow cytometry. Humanized NSG mice were established as described in a previous study [23]. Briefly, 3-week-old NSG mice received sublethal irradiation (360 cGy; X-RAD 320 irradiator), followed by an intrahepatic injection of 1 × 10^5^ human cord blood-derived CD34^+^ cells. Twelve weeks later, human immune cells from the humanized reconstituted NSG mice were analyzed using flow cytometry. Only humanized NSG mice in which human CD45^+^ cells accounted for ≥25% of the total circulating CD45^+^ cells were considered successfully established and used in subsequent experiments.

### Tumor growth assay in vivo

HGC-27 human GC cells were subcutaneously implanted into the right flank of BALB/c nude mice or humanized NSG mice. Fourteen days after the injection of tumor cells, when a measurable tumorous node was palpable, mice were randomized to receive PBS (vehicle control), nab-paclitaxel (10 mg/kg, twice a week) or OTX008 (5 mg/kg, twice a week) via intraperitoneal injection for 6 weeks. The tumor size was measured twice weekly, and the tumor volume (V) was calculated using the formula Volume = 1/2 Length × Width^2^. Tumor nodes were dissected and processed for immunohistochemical staining. The in vivo experiments using mice conformed to the rules of the Animal Ethics Committee of Zhongshan Hospital affiliated with Fudan University.

### Statistical analysis

GraphPad Prism version 8.0 (GraphPad Software Inc., San Diego, CA) was utilized for statistical analyses. Student’s t-test was performed to compare quantitative data between two groups. The correlation between the expressions of two genes was assessed by calculating Spearman’s correlation coefficient. The Kaplan-Meier analysis and log-rank test were employed to analyze overall survival and the cumulative recurrence rate. *P* < 0.05 was considered a statistically significant difference. Illustrations were created with BioRender scientific illustration software through a paid subscription (biorender.com).

## Results

### ZFP64 exhibits upregulated expression in nab-paclitaxel resistant GC

**Fig. 1:**
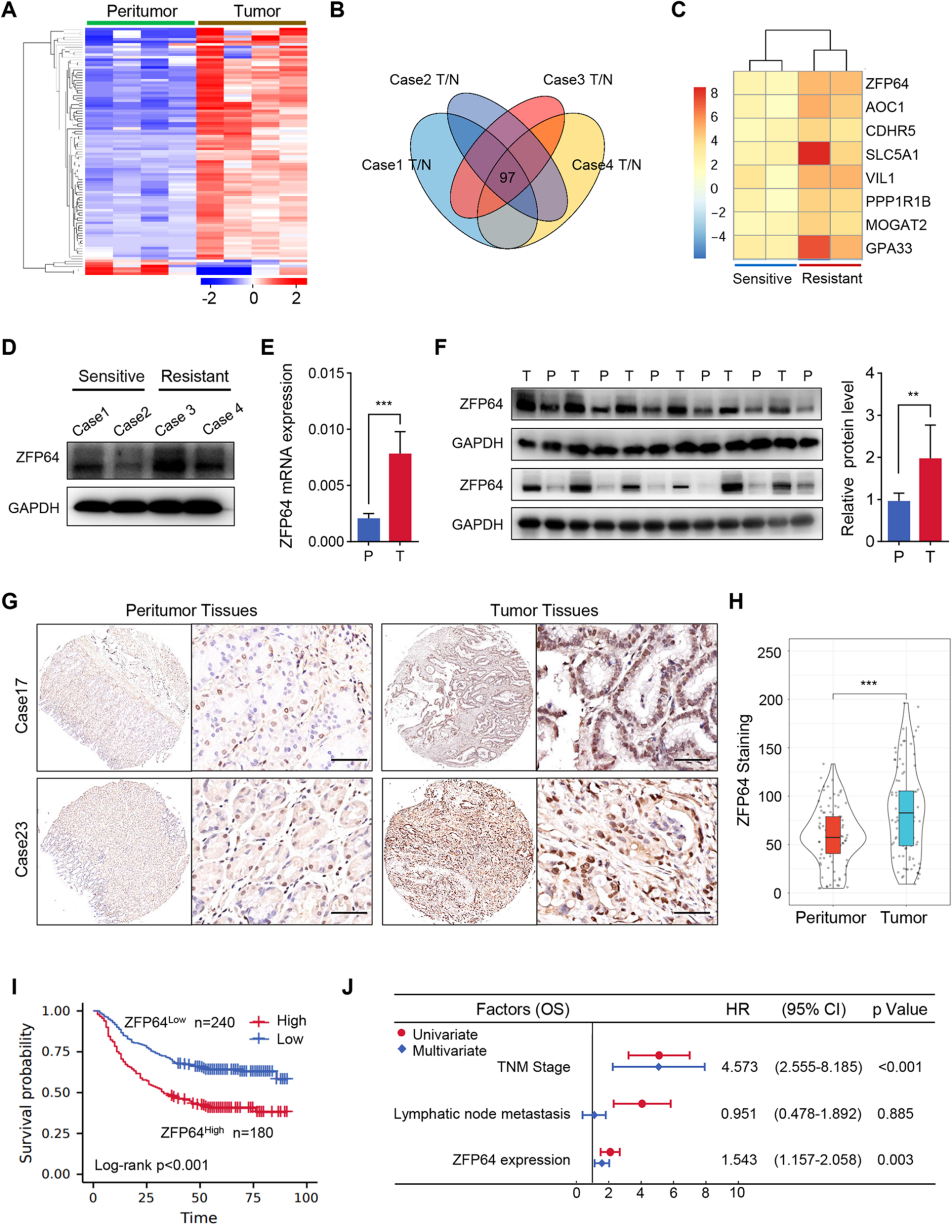
ZFP64 expression is increased in human GC tissues and prominently expressed in nab-paclitaxel-resistant GCs. **A** Heatmap illustrating the differentially expressed genes based on the RNA-sequencing data from GC tissues and surrounding normal tissues. **B** Venn diagram showing the number of overlapping genes that were differentially expressed between four paired GC tissues or surrounding normal tissues. **C** Heatmap illustrating the differentially expressed genes between nab-paclitaxel-sensitive and nab-paclitaxel-resistant GC tissues. **D** ZFP64 levels in nab-paclitaxel-sensitive (*n* = 2) and nab-paclitaxel-resistant (n = 2) GC tissues was examined by western blotting. **E** qRT-PCR analysis of ZFP64 mRNA in GC tissues (T) and corresponding normal tissues (P) from the Zhongshan cohort (*n* = 20). **F** Western blot analysis of paired human GC samples (left panel) and quantified by using ImageJ software (right panel). **G-H** IHC staining for ZFP64 was performed on a human GC tissue microarray (**G**) and quantified by using Image-Pro Plus software (**H**). Scale bars, 50 μm. **I** Overall survival curve for patients with low or high ZFP64 expression (*P* = 0.039). **J** Forest plot of significant factors associated with OS in GC patients. Data are presented as the means ± SEM. **P < 0.01; *** *P* < 0.005. ZFP64, Zinc Finger Protein 64; IHC, immunohistochemistry; GC, gastric cancer; OS, overall survival; qRT-PCR, quantitative reverse transcription PCR

To screen the key genes resulting to nab-Paclitaxel resistance in GC, four paired samples from patients who were resistant or sensitive to AS-based neoadjuvant chemotherapy were collected for RNA-seq analysis. Firstly, differentially expressed genes (DEGs) between tumor and adjacent peritumor samples from 4 GC patients were analyzed. With a threshold (average FPKM ≥5, Fold Chang ≥2 and *P* < 0.05), 97 highly abundant genes were identified significantly upregulated in GC samples compared with the adjacent peritumor samples (Fig. 1A-B), which were validated using qRT-PCR of 15 different genes (Fig. S1A-B, R = 0.910, *P* < 0.0001). We further assessed DEGs between chemotherapy-resistant and chemotherapy-sensitive tumor samples. By overlapping the two DEG panels, 8 genes were found simultaneously upregulated in tumor and chemotherapy-resistant samples (Fig. 1C). Interestingly, some previously reported chemoresistance-related genes, such as P glycoprotein (P-gp), adenosine triphosphate-binding cassette superfamily G member 2 (ABCG2), and multidrug resistant associate protein (MRP) were not found in our analysis results (data not shown), indicating a novel mechanism might be involved in chemotherapy resistance of GC. To validate our findings, we searched the expression profile of 8 genes in TCGA database and found ZFP64 expressed much higher in GC tissues, consistent with our results (Fig. S1C). Therefore, ZFP64 was chosen for further study.

Next, ZPF64 were confirmed significantly upregulated in nab-paclitaxel-resistant GC samples by western blot analysis (Fig. 1D). ZFP64 mRNA and protein expression in 20 pairs of GC and adjacent peritumor tissues were examined (Fig. 1E-F). The IHC staining of ZFP64 in a GC tissues microarray also indicate higher ZFP64 expression in tumors than in peritumor tissues (Fig. 1G-H). Combined with clinical parameters, we found high ZFP64 expression significant correlated with lymph node metastasis *(P* < 0.001), a large tumor size (*P* < 0.001), pTNM stage (*P* < 0.001) and thrombus in vessels encapsulating the tumor (*P* < 0.001) (Table S3). Furthermore, patients with high ZFP64 expression (*n* = 180) had shorter survival times than patients with low ZFP64 expression (*n* = 240, Fig. 1I). The Cox regression analysis indicated ZFP64 expression as an independent prognostic factor for GC patients (Fig. 1J). Collectively, ZFP64 might be a key promoter of nab-Paclitaxel resistance and progression of GC.

### ZFP64 promotes GC cell motility and invasion in vitro

**Fig. 2:**
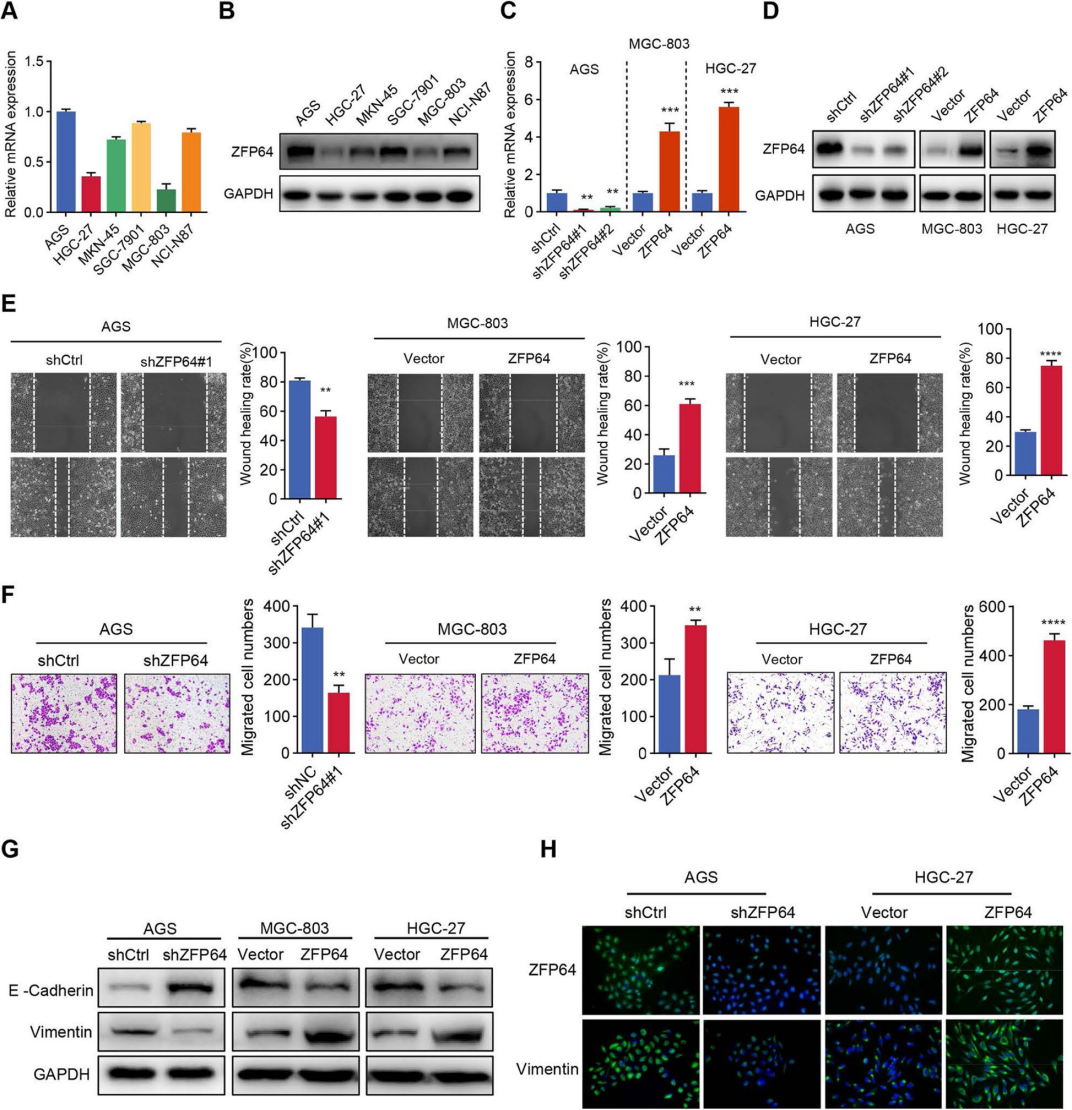
ZFP64 promotes GC cell migration and invasion in vitro. **A-B** The ZFP64 mRNA and protein level were detected in various GC cell lines. **C-D** Successful knockdown of ZFP64 in AGS cells and overexpression of ZFP64 in MGC-803 and HGC-27 cells. **E-F** The migration and invasion of AGS parental cells were compared with ZFP64 knockdown cells; the migration and invasion of MGC-803 or HGC-27 parental cells were compared with ZFP64-overexpressing cells. **G-H** The expression of EMT-related proteins was analyzed in ZFP64 knockdown or overexpressing cells using western blotting and immunofluorescence staining. All experiments were repeated at least 3 times. EMT, epithelial-mesenchymal transition; qRT-PCR, quantitative PCR with reverse transcription. ** *P* < 0.01, *** *P* < 0.005, and **** *P* < 0.001

Given the clinical evidence suggesting a potential role for ZFP64 in tumor metastasis, we sought to experimentally confirm this possibility. We first analyzed the expression of ZFP64 in 6 GC cell lines (Fig. 2A-B). ZFP64 was then stably knocked down in AGS cells with high ZFP64 expression, while ZFP64 was stably overexpressed in HGC-27 and MGC-803 cells with low ZFP64 expression (Fig. 2C-D). Wound-healing and invasion assays showed that ZFP64 knockdown significantly decreased the migration and invasion of AGS cells. Conversely, overexpression of ZFP64 promoted the metastatic activity of MGC803 and HGC27 cells (Fig. 2E-F). Furthermore, we analyzed markers associated with EMT and found cells with elevated ZFP64 levels expressed higher vimentin, but lower E-cadherin (Fig. 2G-H). Thus, the upregulation of ZFP64 promotes GC cell invasion and metastasis in vitro.

### ZFP64 reduces sensitivity to nab-paclitaxel treatment in GC both in vitro *and* in vivo

**Fig. 3:**
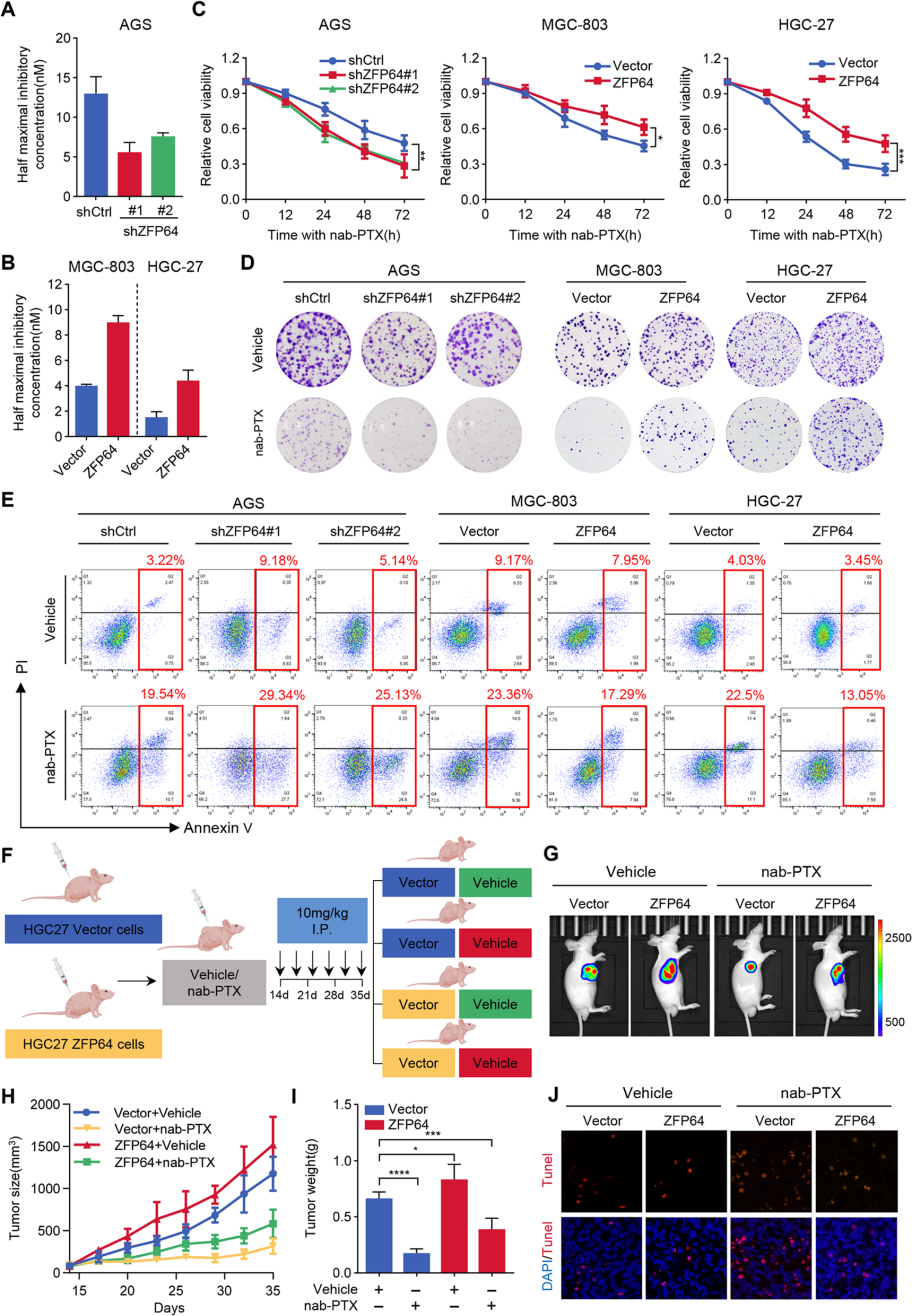
ZFP64 reduced the sensitivity of gastric cancer cells to nab-paclitaxel. **A**. The IC50 value of control and ZFP64-overexpressing AGS cells were measured after treated with nab-paclitaxel for 72 h. **B** The IC50 value of control and ZFP64-knockdown GC cells after treatment for 72 h. **C** GC cells were cultured in media and treated with nab-paclitaxel (AGS, 15 nM; MGC-803, 5 nM; HGC-27, 5 nM). After 12, 24, 48 or 72 h of treatment, cell viability was determined by CCK8 assays (*n* = 6). **D** GC cells growing in 6-well plates (50,000 cells/well) were exposed to vehicle or nab-paclitaxel (AGS, 15 nM; MGC-803, 5 nM; HGC-27, 5 nM) for 24 h. The cells were trypsinized and collected. One thousand cells from each treatment group were reseeded in 6-well plates. A clonogenic assay was performed. The plates were photographed under a light microscope. **E** GC cells were grown in duplicate wells of 6-well plates and exposed to vehicle or nab-paclitaxel for 48 h. At the end of the treatment period, cells were trypsinized, and cell apoptosis was evaluated using flow cytometry and an Annexin V/PI Apoptosis Detection Kit. **F** Schematic representation of the experimental procedure. HGC27 vector cells or HGC27 ZFP64 cells (2 × 10^6^) were subcutaneously injected into athymic nude mice. Fourteen days later, mice were intraperitoneally injected with either vehicle or 10 mg/kg nab-paclitaxel for 3 weeks (twice a week, 8 mice/group). **G** Representative bioluminescence images of xenografts from mice treated with vehicle or nab-paclitaxel. **H-I** Tumor growth curves and tumor weights of different treatment groups were analyzed. **J** TUNEL staining in FFPE samples of tumors from the indicated treatment groups. All experiments in vitro were repeated for 3 times. Data are presented as the means ± SEM. * *P* < 0.05; ** *P* < 0.01; *** *P* < 0.005; **** *P* < 0.001. FACS, fluorescence-activated cell sorting; TUNEL, terminal-deoxynucleotidyl transferase-mediated nick end labeling; DAPI, 4–6-diamidino-2-phenylindole

We then asked if ZFP64 altered nab-paclitaxel sensitivity. For this, we treated cells with a range of nab-paclitaxel doses and calculated the half-maximal inhibitory concentration (IC50) of nab*-*paclitaxel in different group cells (Fig. S2A, Fig. 3A-B). Knockdown of ZFP64 increase cell viability and growth when treated with nab-paclitaxel, in CCK8 and long-term colony formation assays respectively, while ZFP64 overexpression caused converse results (Fig. 3C-D). Additionally, ZFP64 inhibited cell apoptosis induced by nab*-*paclitaxel (Fig. 3E, Fig. S2B). Moreover, mouse xenograft models were next established to validate above findings in vivo (Fig. 3F, Fig. S5A). As shown in Fig. 3G-I, nab-paclitaxel treatment elicited potent inhibition of tumor growth; however, the growth inhibition caused by nab-paclitaxel was diminished in ZFP64-overexpressing tumors. TUNEL staining consistently indicated ZFP64 overexpression reduced nab-paclitaxel-induced apoptosis in vivo (Fig. 3J). Furthermore, we also found that GC cells with high level of ZFP64 resisted to 5-Fu, Cisplatin, Oxaliplatin and Irinotecan (Fig. S3). Based on these results, we concluded that high level of ZFP64 decreased the therapeutic efficiency of nab*-*paclitaxel in GC.

### Elevated ZFP64 confers a stem-like phenotype to GC cells

**Fig. 4:**
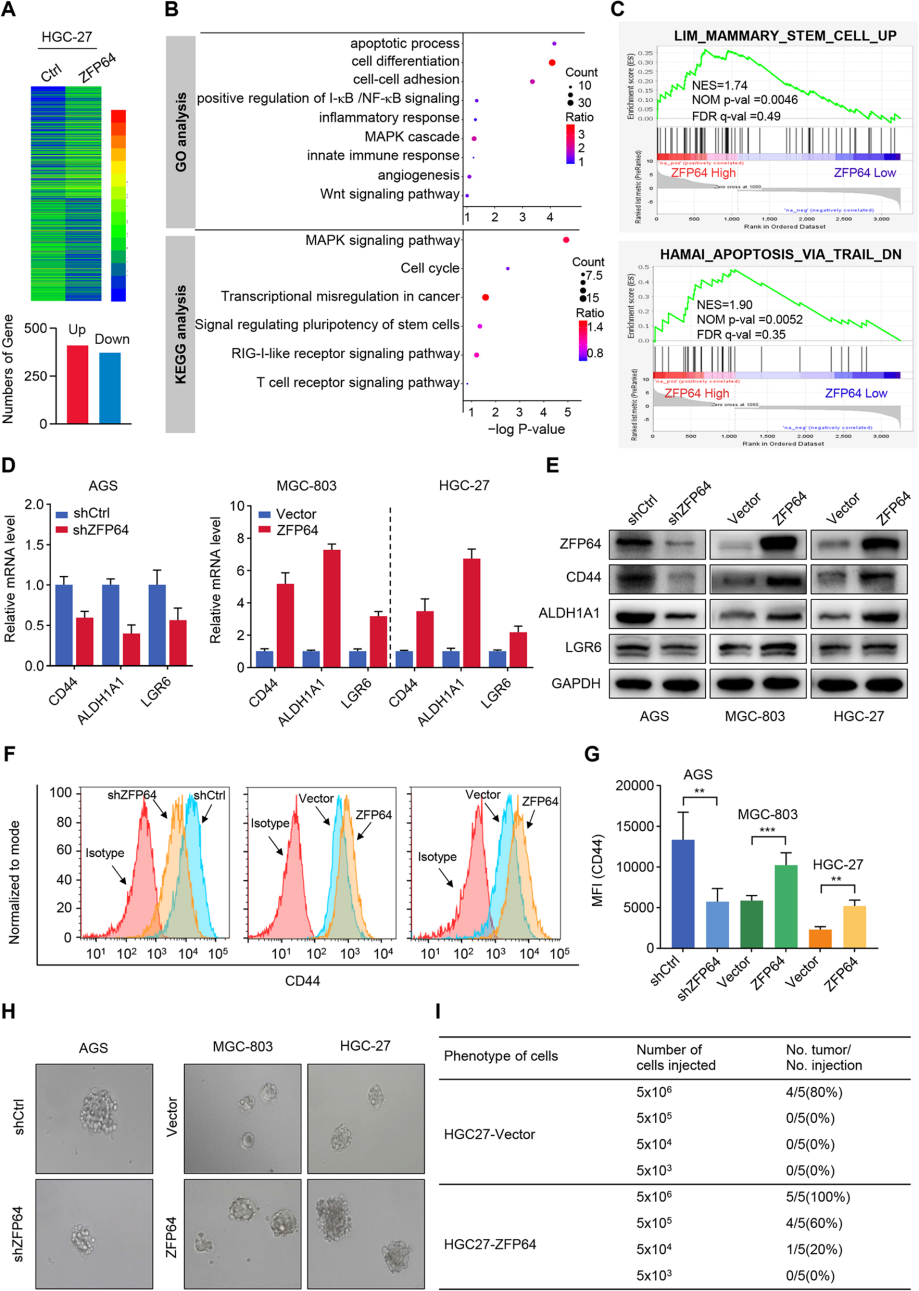
ZFP64 mediated stemness enhancement in GC cells. **A** Heatmap showing changes in gene expression after the overexpression of ZFP64 in HGC-27 cells. **B** The enrichment of KEGG pathways and Gene Ontology (GO) terms among differentially expressed genes was determined by the DAVID Functional Annotation tool. **C** The GSEA revealed a significant enrichment of the upregulated stem cell signature gene set and downregulated apoptosis signature gene set in ZFP64-overexpressing HGC-27 cells. **D-E** qRT-PCR and western blot showing the levels of stemness-related genes in GC cells with different ZFP64 expression levels. **F-G** Levels of the stemness-related protein CD44 in GC cells were analyzed using flow cytometry. Blue represents the isotype control. **H** Sphere forming assay of GC cells with ZFP64 knockdown or overexpression. **I** ZFP64 overexpression enhanced tumor-initiating capacity in vivo, as analyzed by a limiting dilution assay. Data are presented as the means ± SEM. ** *P* < 0.01; *** *P* < 0.005

To better understand the roles of ZFP64 in GC development, we evaluated global gene expression in control and ZFP64 overexpression HGC27 cells. As shown in Fig. 4A, 410 genes were upregulated and 372 genes were downregulated in ZFP64 overexpression cells. Among all the DGEs, 10 genes were randomly selected to validate the RNA-seq results by performing qRT-PCR with high confidence (Fig. S2C-D). GO and KEGG enrichment analysis showed these DEGs were mainly involved in “apoptotic process”, “cell differentiation”, “Cell cycle” and “MAPK signaling pathway” (Fig. 4B). Consistently, GSEA analysis revealed that ZFP64 levels was correlated to cell stemness and resistance to apoptosis (Fig. 4C). Given that stemness-like activation often contributes chemoresistance in cancer, we hypothesized that ZFP64 may also promote gastric cancer stemness.

To investigate this possibility, the expression levels of some stemness-related genes were detected in ZFP64 knockdown or overexpression cells. As expected, ZFP64 depletion significantly reduced the expression of CD44, ALDH1A1 and LGR6 in AGS cells, whereas ZFP64 overexpression promoted their expression in HGC27 and MGC803 cells (Fig. 4D-E). In addition, flow cytometry analysis further confirmed that ZFP64 depletion or overexpression respectively decreased or increased CD44^+^ populations in GC cells (Fig. 4F-G). Consistently, spheroid assays showed that ZFP64 shRNA significantly impaired the spheroid-formation ability in AGS cells, whereas ZFP64 overexpression greatly enhanced it in HGC27 and MGC803 cells (Fig. 4H). More importantly, limiting dilution assays further confirmed the pro-stemness ability of ZFP64, because HGC27 cells with ZFP64 overexpression showed enhanced tumor initiation capability (Fig. 4I). Taken together, these findings strongly indicate that ZFP64 can induce stem-like phenotype in GC cells.

### ZFP64 transcriptionally activates GAL-1 in GC cells

**Fig. 5:**
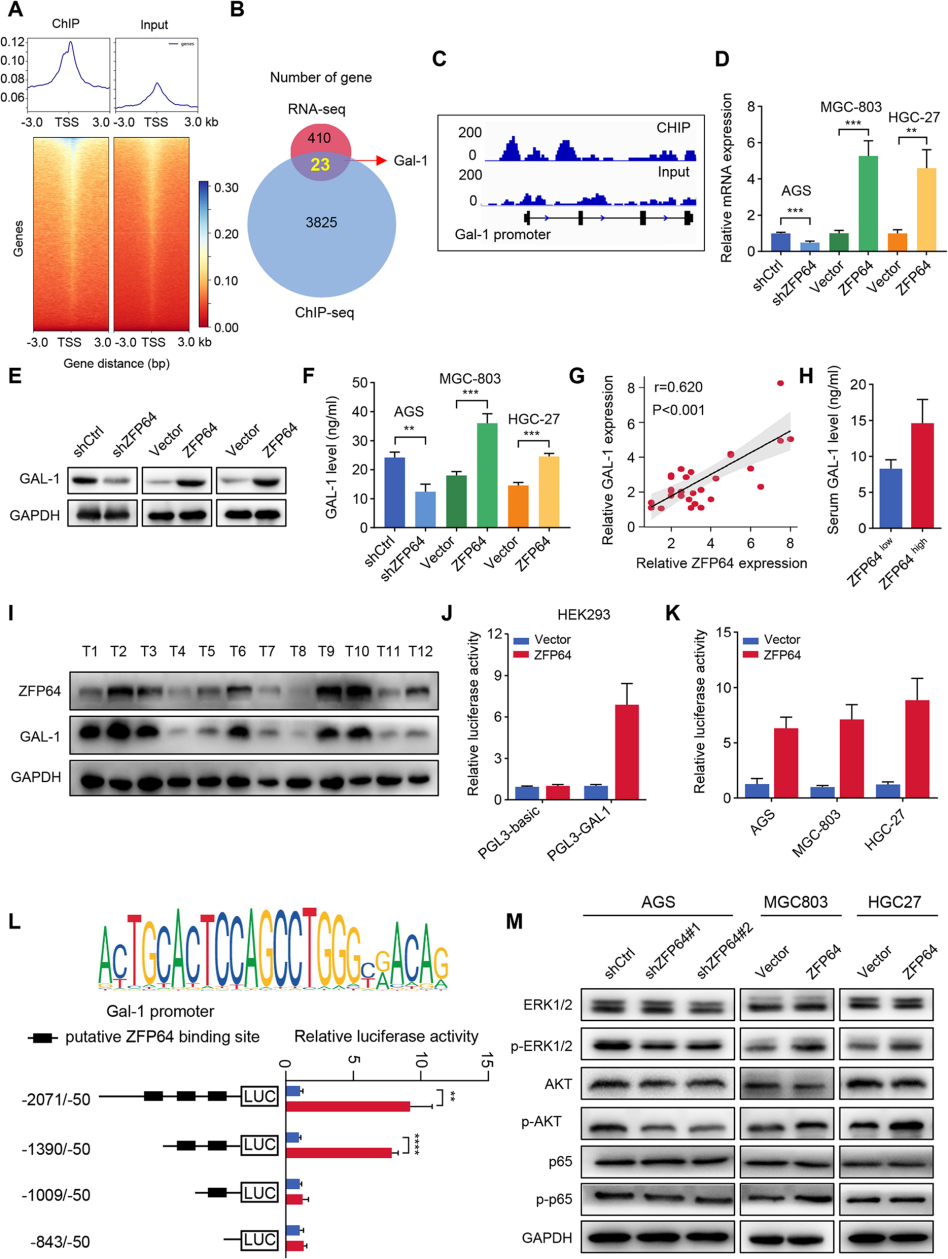
The transcription factor ZFP64 regulates the transcription of GAL-1 in GC cells. **A** Heatmaps displaying ZFP64-binding sites in a 3 kb window around chromatin immunoprecipitation site followed by ChIP-seq peak summits in HGC-27 cells. **B** Overlap between RNA-seq and ChIP-seq data. **C** Representative browser track images showing the intensity of the ChIP-seq signal for GAL-1 in HGC-27 cells compared with the input control. **D-E** GAL-1 mRNA and protein were detected in GC cells with different ZFP64 expression levels. **F** The culture supernatants of GC cells were collected and analyzed for GAL-1 levels by ELIASA assay. **G** Pearson’s correlation analysis of the expression of ZFP64 and GAL-1 in GC tissues. **H** Serum levels of the GAL-1 protein in patients with GC. **I** Western plot analysis of ZFP64 and GAL-1 in GC tissues. **J** Relative luciferase reporter assays in HEK293 cells co-transfected with plasmid constructs containing the GAL-1 promoter with a ZFP64-overexpressing construct or control construct. **K** Relative luciferase reporter assays in the AGS, HGC-27, and MGC-803 cell lines after cotransfection of plasmid constructs containing the GAL-1 promoter with a ZFP64-overexpressing construct. **L** ZFP64-binding motif (upper panel); Serially truncated GAL-1 promoter constructs were transfected into HGC-27 cells. Then, pCMV-ZFP64 plasmids were co-transfected, and a luciferase reporter assay was conducted (lower panel). **M** ZFP64 active ERK1/2 and AKT signals, but have no effect on phosphorylation of p65. Data are presented as the means ± SEM. ** *P* < 0.01; *** *P* < 0.005

To probe the molecular mechanism of nab-paclitaxel resistance and enhanced stem-like phenotype, we searched the downstream target genes of transcript regulator ZFP64 (Fig. 5A). ChIP sequencing were performed in ZFP64-overexpression cells and identified 36,966 high-confidence binding sites in 9330 genes (Fig. S4). Notably, 5846 binding sites were found in the promoter regions of genes, among which 12.35% were located within a distance ≤1 kb, 3.65% were located within 1–2 kb, and 2.87% were located within 2–3 kb from transcription start sites (TSS) (Fig. S4A-B).

By overlapping the 3825 genes containing ZFP64-binding sites within the distance of ≤1 kb and 410 differentially expressed genes between control and ZFP64 overexpression HGC27 cells, 23 genes were obtained (Fig. 5B-C). GAL-1 stood out immediately from this list, as it has been previously reported to endow cancer cells with CSC phenotypes and crippling anticancer immunity.

The relation between ZFP64 and GAL-1 expression were then detected. qRT-PCR and western blotting indicated that cells with elevated ZFP64 expressed high level of GAL-1 in GC cells (Fig. 5D-E). Because GAL-1 is a secreted protein, we also examined the GAL-1 levels in culture supernatant of GC cells by ELISA assay and obtained similar results (Fig. 5F). In addition, ZFP64 and GAL-1 expression in GC tissues were also positively correlated (Fig. 5G-I). Consistent with these findings, serum GAL-1 levels in were also higher in patients with high levels of ZFP64 than in patients with low levels of ZFP64 (Fig. 5H). To illustrates the regulatory relationship between ZFP64 and GAL-1, HEK293 cells were transfected with pGL3-GAL-1/ pGL3-basic with or without the ZFP64 plasmid. As shown in Fig. 5J, ZFP64 significantly increased the activity of the GAL-1 promoter compared with the transfected control in the pGL3-GAL-1 promoter group, but not in the pGL3-basic group. Similar results were also observed in AGS, HGC27 and MGC803 cells (Fig. 5K). To further determine the core activating region, we first scanned for ZFP64 binding motif using FIMO (Find Individual Motif Occurrences), part of the MEME Suite, and identified three putative binding sites (− 866 to − 843, − 1032 to − 1009 and − 1413 to − 1390). Next, four fragments of GAL-1 promoter region were cloned and inserted into the pGL3-basic vector, dual-luciferase reporter assay showed that the fragment (− 1390 to − 1009) was necessary for ZFP64-medaited transcriptional activation (Fig. 5L). By constructing mutated plasmids, we found the mutation of putative binding sites (− 1032 to − 1009) attenuated the ZFP64-mediated enhancement of GAL1 promoter reporter activity, indicating that this region is the directly binding site of ZFP64 (Fig. S4C). These results demonstrated that ZFP64 regulated GAL-1 expression by inducing GAL-1 promoter activation at the transcription level.

Finally, considering GAL-1 has been reported to active MAPK and PI3K/AKT signals, we validated our previous functional analysis of RNA-seq to scrutinize the downstream intermediates in the mechanism by which ZFP64 promotes GC progression. Core genes in NF-κB, MAPK and PI3K signaling pathways were detected in cells with different levels of ZFP64 expression. We observed higher levels of phosphorylated ERK1/2 and AKT in GC cells overexpressing ZFP64 than in cells with low levels of ZFP64, while the level of phosphorylated p65 showed no obvious difference (Fig. 5M). Together, these results indicate ZFP64 promotes the transcriptional activation of GAL-1 and actives MAPK and PI3K/AKT signaling pathway.

### Elevated ZFP64 induces compromises anticancer immune responses in GC

**Fig. 6:**
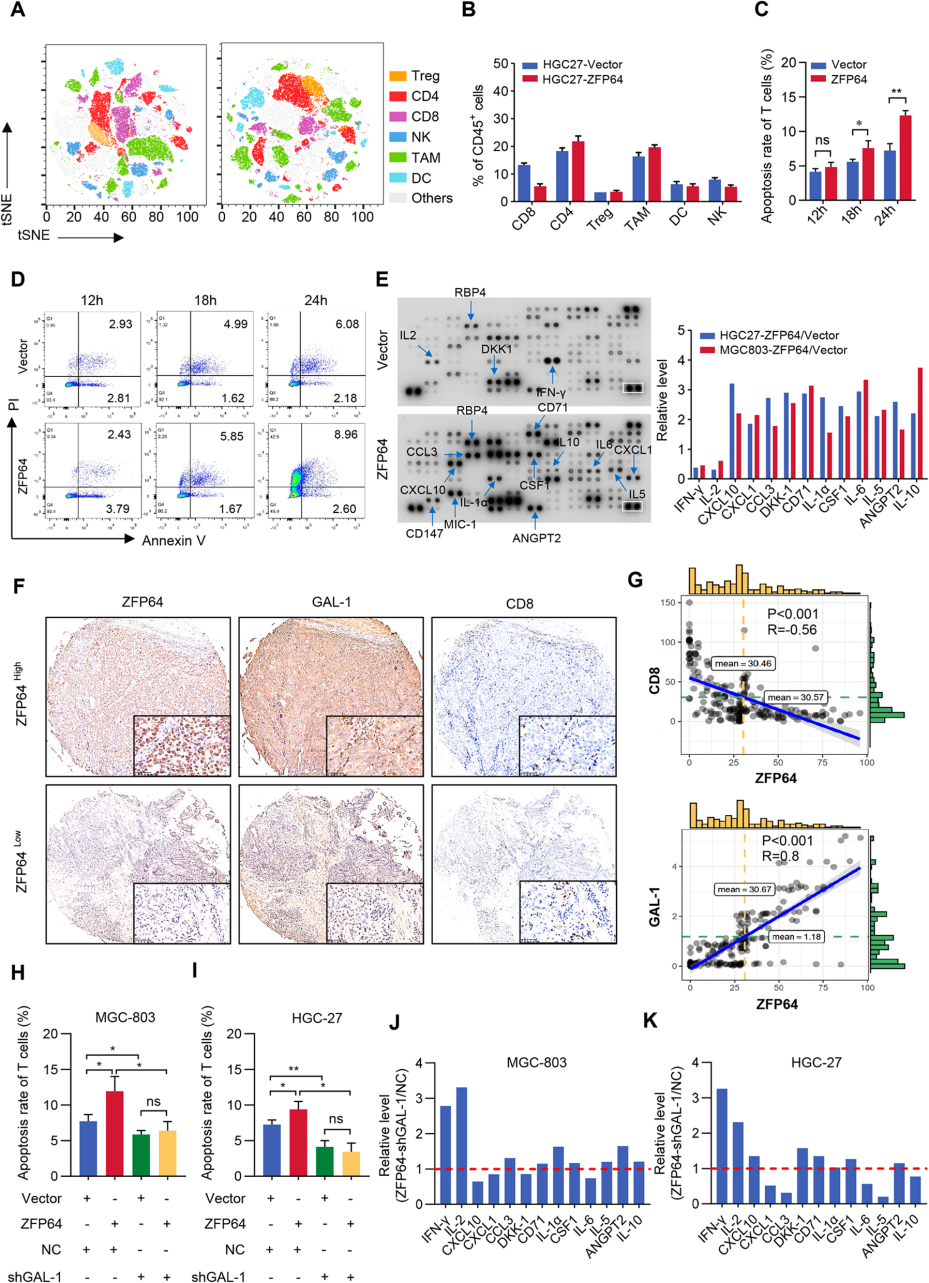
ZFP64 induces immune microenvironment changes. **A**-**B** CyTOF analysis and quantification of immune cell populations was performed in humanized NSG mice with different ZFP64 expression. **C-D** T cells were cocultured with HGC-27 cells, and the apoptosis rate of T cells was assayed using Annexin V/PI staining in combination with FACS analysis. **E** An inflammatory cytokine antibody array was used to assess cytokine secretion from HGC-27/MGC-803-ZFP64 cells, control cells and blood lymphocyte coculture supernatants. The left panel shows the cytokine antibody array incubated with the supernatant of HGC-27-ZFP64/control cells and blood lymphocyte coculture. Data are presented as the gray ratio between two groups. **F** Representative images of immunohistochemical staining for ZFP64, GAL-1 and CD8 in the TMA of gastric cancer. **G** Pearson’s correlation analysis of the expression of ZFP64 and CD8 in TMA (upper panel) and ZFP64 and GAL1 expression in TMA (lower panel). **H-I** The increase in T cell apoptosis induced by ZFP64 overexpression in GC cells was abrogated by GAL-1 knockdown. **J-K** An inflammatory cytokine antibody array was used to assess the changes in cytokine levels between HGC-27/MGC-803-ZFP64-shGAL-1 cells and their control cells cocultured with blood lymphocytes. Data are presented as the means±SEM. * *P* < 0.05; ** *P* < 0.01

Since RNA-sequence analysis suggests ZFP64 might involve in the inflammatory response and innate immune response, and GAL-1 functions as a negative regulator of innate immune cells, including T cell activation and survival, and results in immune evasion of tumor cells [24], we screened the impact of tumors with high level of ZFP64 on the tumor immune microenvironment in the humanized NSG mice by CyTOF. Thus, we collected subcutaneous xenotransplanted tumors from humanized NSG mice to measure the immune cell composition in CD45^+^ tumor infiltrating leukocytes (TIL) by flow cytometry and found that CD8^+^ T is significantly lower in tumors with high level of ZFP64 than that in tumors with low level of ZFP64. Moreover, the TAM and Treg showed a slightly upregulation in tumors with high level of ZFP64 compared to the control (Fig. 6A-B). Here, we further established a coculture system of peripheral blood mononuclear cells (PBMCs) and tumor cells with different levels of ZFP64 expression. As shown in Fig. 6C-D, ZFP64-overexpressing HGC-27 cells induced apoptosis in a significantly greater percentage of CD8^+^ T cells than in control cells at different time points. Furthermore, human cytokine arrays were used to detect the cytokine profile in coculture supernatants, and elevated ZFP64 expression was associated with low levels of the effector cytokines IFN-γ and IL-2 and high levels of immunosuppressive cytokines such as CXCL10, CXCL1, CCL3, DKK1, CD71, IL-1α, CSF1 and IL-6 in the supernatants (Fig. 6E). Moreover, we confirmed the relationships among the expression of ZFP64, GAL-1 and CD8 in TMA (Fig. 6F-G). Importantly, the apoptosis of CD8^+^ T cells were reversed by interfering the GAL-1 expression in GC cells overexpressing ZFP64 (Fig. 6H-I). Accordingly, we also found the levels of immunosuppressive cytokines endowed by high level of ZFP64 was impaired by GAL-1 interference (Fig. 6J-K). Thus, we concluded that ZFP64 overexpression induced a compromised immunosuppressive microenvironment in GC.

### GAL-1 interference ablates the CSC phenotype induced by elevated ZFP64 levels

**Fig. 7:**
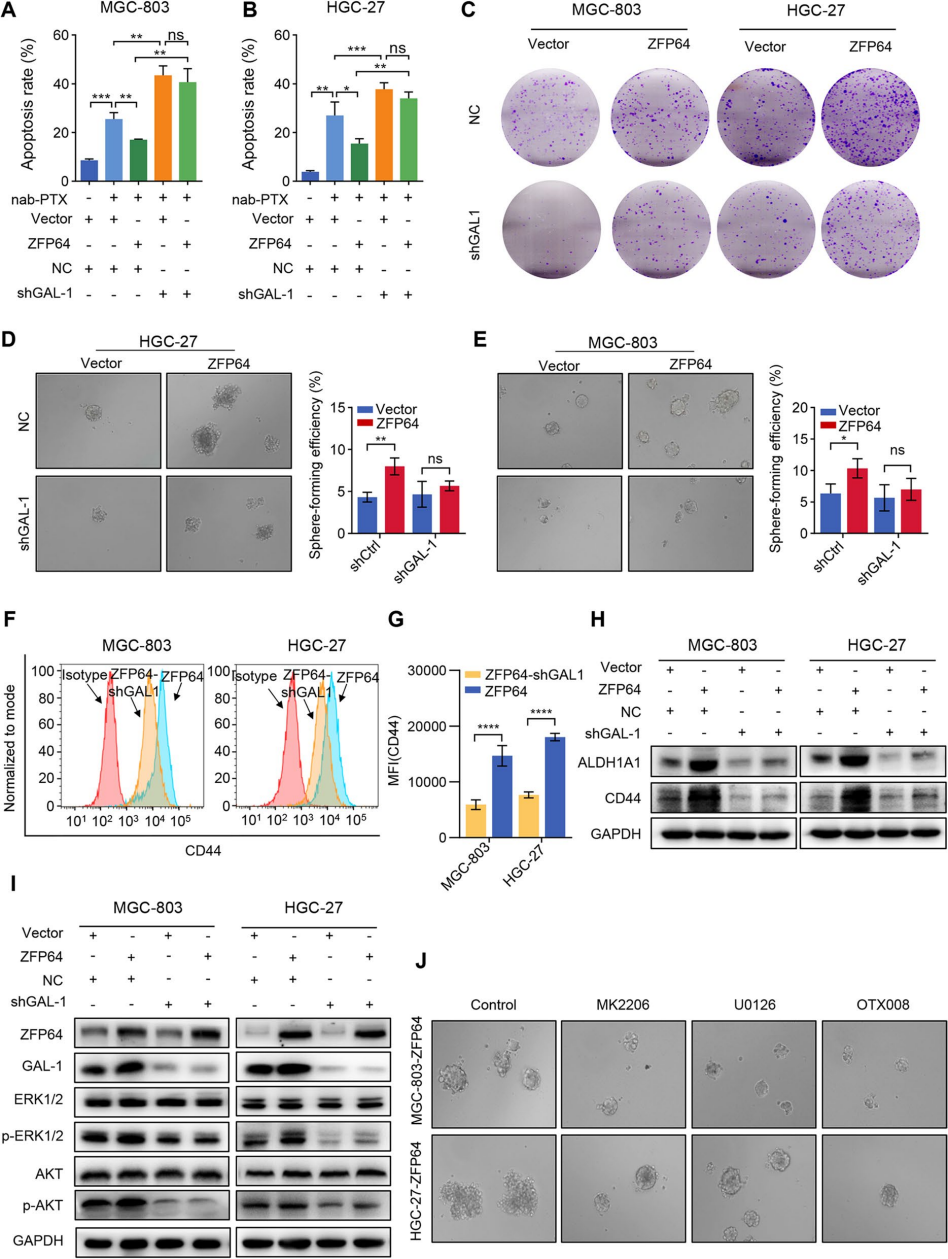
Knockdown of GAL-1 rescues CSC-like properties induced by ZFP64 overexpression in GC cells. **A-B** Knockdown of GAL-1 diminished the inhibitory effect of ZFP64 on nab-paclitaxel-induced apoptosis. HGC-27 and MGC-803 cells overexpressing ZFP64 and control cells were transfected with NC or shGAL-1 for 24 h and then treated with nab-paclitaxel for 48 h before the apoptosis rate was examined. **C** Knockdown of GAL-1 increased the sensitivity of nab-paclitaxel in ZFP64 overexpression cells. **D-E** The enhanced sphere formation ability induced by ZFP64 overexpression in GC cells was abrogated by GAL-1 silencing. **F** High CD44 level in ZFP64-overexpression cells was decreased by GAL-1 silencing. **G** Western blot analysis of the activation of ERK and AKT signaling after the co-transfection of shGAL-1 and ZFP64 overexpression constructs into GC cells. **H** ERK inhibitor (U0126) 、AKT inhibitor (MK2206) and GAL-1 inhibitor (OTX008) inhibited sphere formation of ZFP64 overexpressing GC cells. Data are presented as the means±SEM. * P < 0.05; ** P < 0.01; *** *P* < 0.005; **** *P* < 0.001

Here, we further determined the role of GAL-1 in ZFP64-induced GC with stem cell properties progression. Firstly, we showed that the apoptotic rate of GC cells induced by nab-paclitaxel increased substantially after GAL-1 interference (Fig. 7A-B), and the ability of clone formation conferred by ZFP64 overexpression was inhibited by GAL-1 interference (Fig. 7C). Moreover, the sphere formation was significantly impaired upon GAL-1 silencing in cells with high levels of ZFP64 (Fig. 7D-E). As GC stem cell markers, CD44 and ALDH1A1 were downregulated by interfering the GAL-1 expression in GC cells overexpressing ZFP64.

Then, we analyzed the impact of the GAL-1 interference on the activity of the MAPK, and PI3K/AKT signaling pathways, and found that the downregulation of GAL-1 obviously reduced the levels of phosphorylated ERK1/2 and AKT in cells with high ZFP64 expression, but had no effect on the level of phosphorylated p65 (Fig. 7I). Moreover, we employed MK2206 (an AKT inhibitor) and U0126 (25 μmol/L, an ERK1/2 inhibitor) alone or in combination to observe the colony formation ability of GC cells with different levels of ZFP64 expression. The simultaneous inhibition of ERK1/2 and AKT by OTX008 (an GAL-1 inhibitor) visibly reduced sphere formation, while the inhibition of ERK1/2 or AKT alone only slightly changed the number of colonies formed (Fig. 7J). Based on the results of the experiments described above, we concluded that ZFP64 promoted GC development via GAL-1 transcriptional activation. 

### The combination of OTX008 and nab-paclitaxel is a potent and synergistic therapy

**Fig. 8:**
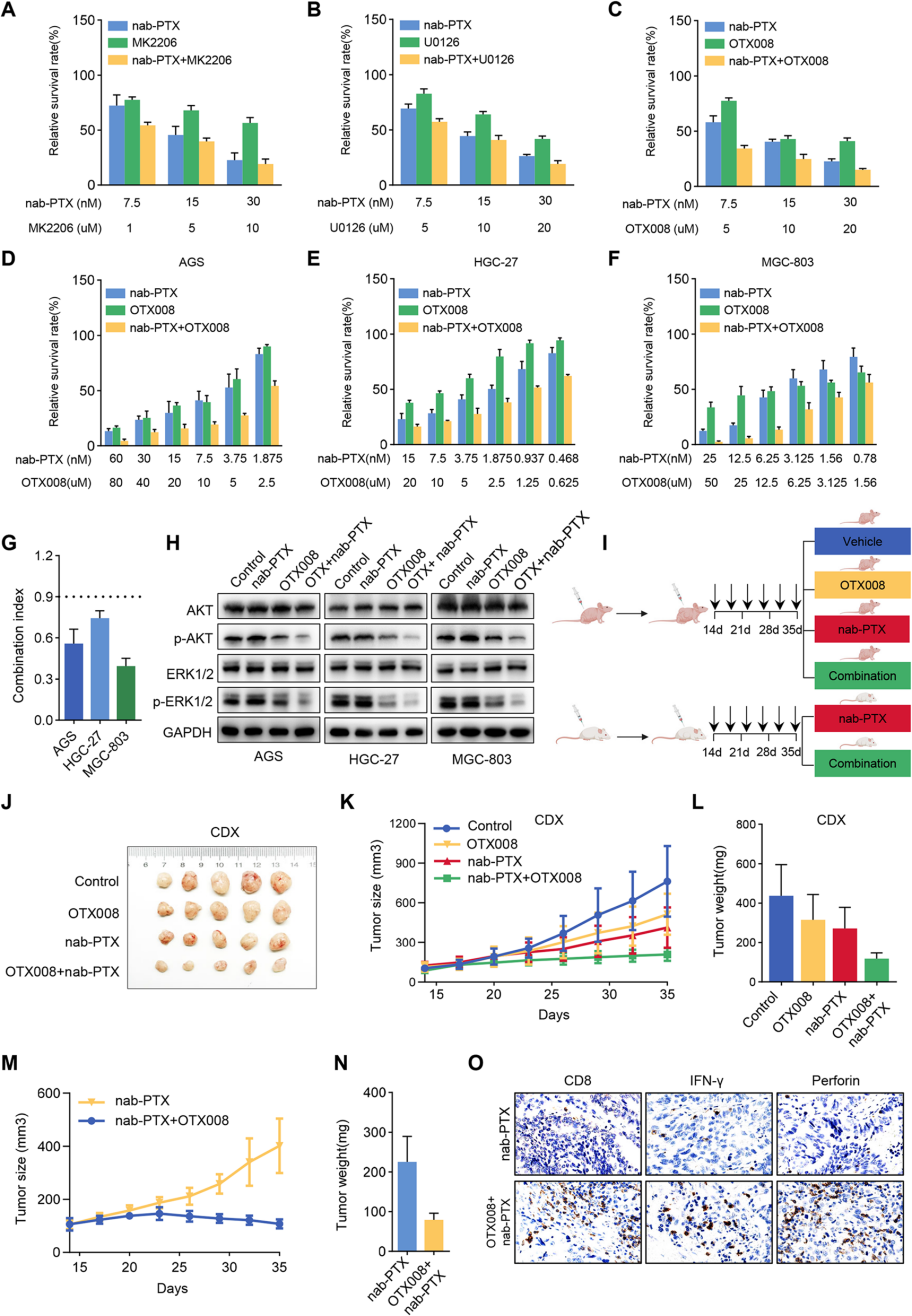
OTX008 alone and OTX008/nab-paclitaxel exert antitumor effect on the GC xenograft model. **A** The cytotoxicity of nab-paclitaxel alone, the AKT inhibitor MK2206 alone, and the combination of nab-paclitaxel and MK2206 in AGS cells. **B** The cytotoxicity of nab-paclitaxel alone, the ERK inhibitor U0126 alone, and the combination of nab-paclitaxel and U0126 in AGS cells. **C** The cytotoxicity of nab-paclitaxel alone, the GAL-1 inhibitor OTX008 alone, and the combination of nab-paclitaxel and OTX008 in AGS cells. **D-F** The cytotoxicity of nab-paclitaxel alone, the GAL-1 inhibitor OTX008 alone, and the combination of nab-paclitaxel and OTX008 in AGS, MGC-803 and HGC-27 cells. **G** The combination index (C.I.) of the treatment with the combination of OTX008 and nab-paclitaxel was calculated using CalcuSyn software. C.I. < 0.9 indicates synergism, C.I. = 0.9–1.10 indicates an additive interaction, and C.I. > 1.1 indicates antagonism. **H** Phospho-ERK and phospho-AKT levels were reduced by the combination treatment (OTX008 and n nab-paclitaxel). **I** Schematic showing the results of the in vivo experiment. ZFP64-overexpressing HGC27 cells (1 × 10^6^) were subcutaneously injected into athymic nude mice (8 mice/group) or humanized mice (5 mice/group). After 2 weeks, mice were treated with vehicle, nab-paclitaxel, OTX008 or OTX008/nab-paclitaxel. After euthanasia, the tumor tissues collected from humanized mice were subjected to immunohistochemical staining. **J** Representative images of tumors from nude mice after various treatments. **K-L** Tumor growth curves and tumor weights of each group. **M-N** Tumor growth curves and tumor weights of humanized mice in different groups. **O** Representative images of IHC staining showing tumor-infiltrating CD8+ lymphocytes, IFN-γ and perforin in serial sections of tumors from humanized mice. Data are presented as the means ± SEM. * *P* < 0.05; ** *P* < 0.01; *** *P* < 0.005

As described above, ZFP64 activated MAPK and AKT signaling pathways in a GAL-1-dependent manner and thus regulated the sensitivity of GC cells to nab-paclitaxel. Here, we further explored the clinical translation of these findings. We examined the synergistic effects of nab-paclitaxel, the AKT inhibitor MK2206, ERK1/2 inhibitor U0126 and GAL-1 inhibitor OTX008 in vitro. According to our combination studies, MK2206 or U0126 sensitized AGS cells to nab-paclitaxel to a limited extent, and OTX008, which simultaneously inhibited the MAPK and PI3K signaling pathways, displayed significant synergistic effects with nab-paclitaxel (Fig. 8A-C). The synergistic effect was further confirmed by measuring the combination index (CI) in the three GC cell lines (Fig. 8D-G). The combination of nab-paclitaxel and OTX008 also exerted synergistic effects on the inhibition of signaling pathways (Fig. 8H).

Based on the aforementioned in vitro data, we established subcutaneous and orthotopic xenograft tumor models with HGC27 cells to determine the effects of OTX008 and nab-paclitaxel in vivo. As shown in Fig. 8I-K, the tumor size and weight significantly decreased after treated with OTX008 or nab-paclitaxel. The tumor response was much more pronounced in the combination treatment group. Similar results were also observed in orthotopic xenograft tumor models (Fig. S5B). Considering the involvement of ZFP64 in the microenvironment, we further compared HGC27 tumor growth in humanized mice treated with nab-paclitaxel monotherapy or combination therapy. Consistent with the results described above, the combination therapy improved the antitumor efficacy compared with nab-paclitaxel monotherapy (Fig. 8L). Importantly, combination therapy was more effective in humanized NSG mice than in immunodeficient SCID mice. Tissue sections from humanized mice contained fewer tumor-infiltrating CD8^+^ lymphocytes and displayed stronger IFN-γ and perforin staining in the combination treatment group (Fig. 8M). Taken together, our findings indicated that a novel therapeutic regimen combining nab-paclitaxel and OTX008 was an efficacious treatment for gastric cancer.

## Discussion

**Fig. 9:**
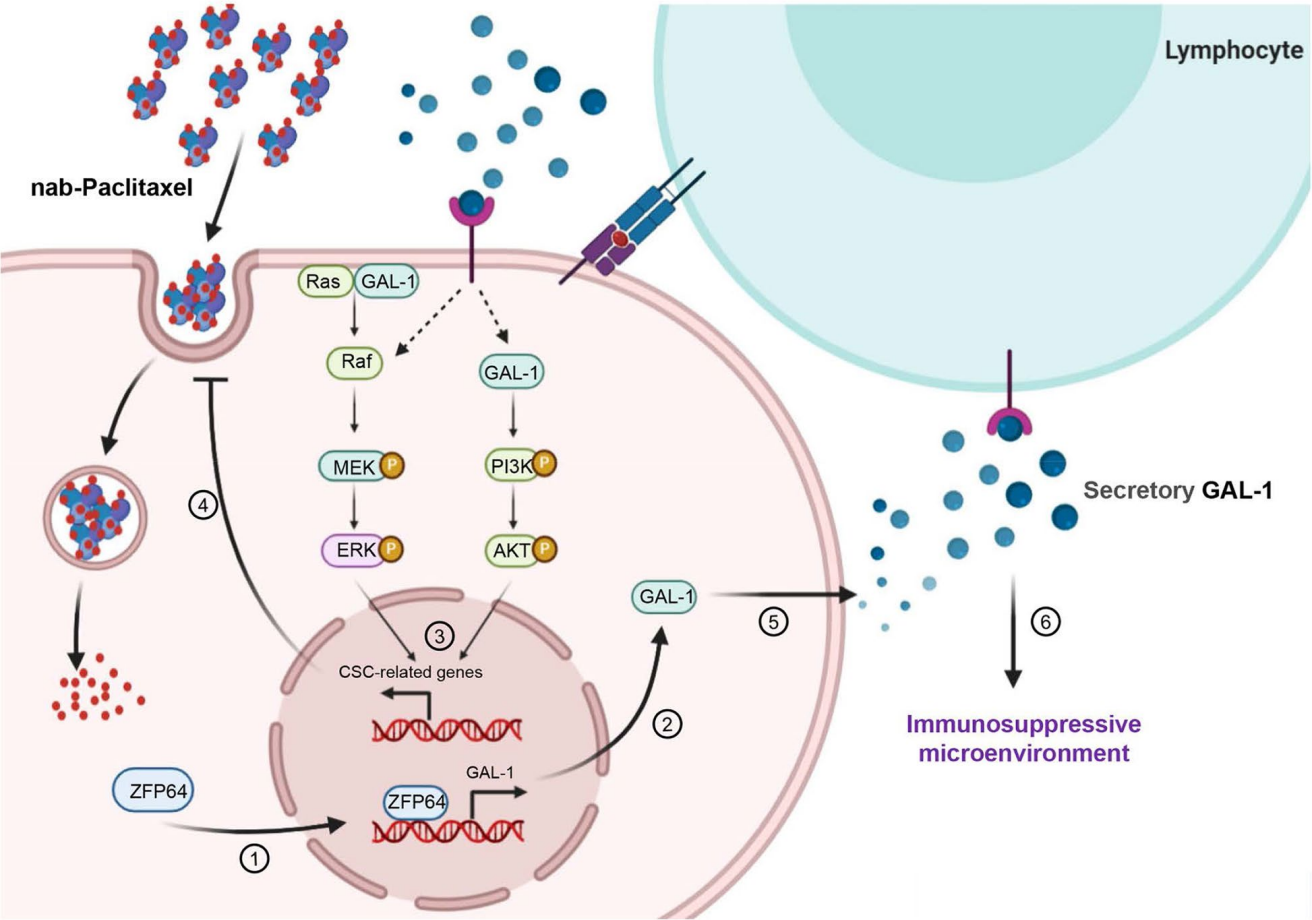
Model depicting the mechanism by which ZFP64 induced nab-paclitaxel insensitivity through the targeting of GAL-1. In gastric cancer cells, ZFP64 overexpression promoted the transcription of GAL-1. Increased intracellular GAL-1 levels induce CSC-like properties by activating the MAPK and PI3K/AKT pathways and subsequently inhibiting the uptake of nab-paclitaxel. Meanwhile, GAL-1 was secreted into the extracellular milieu and stimulated T cells to finally induce an immunosuppressive microenvironment. ① Nuclear translocation of ZFP64, ② the expression of GAL-1; ③ GAL-1 activated the MAPK and PI3K signal to induce the stem cell phenotype of GC cells; ④ the stem cell phenotype resulted in decreased endocytosis of chemotherapy drugs; ⑤ the secretion of GAL-1; ⑥ a high level of GAL-1 promoted an immunosuppressive microenvironment

Chemotherapy resistance is still a major barrier to achieving effective GC treatment. The present manuscript describes very noteworthy findings that may facilitate the development of an efficacious therapy for patients with GC, particularly patients with locally advanced or metastatic cancers. Using RNA-seq, ZFP64 was overexpressed in GC specimens compared with adjacent nontumor specimens, and a significant increase in ZFP64 expression was observed in GC tissues from patients who were resistant to AS-based neoadjuvant chemotherapy compared to patients who were sensitive to AS-based neoadjuvant treatment. Clinically, patients expressing high levels of ZFP64 showed a decreased OS compared with patients with low ZFP64 expression. Importantly, the Cox analysis indicated that ZFP64 expression was an independent prognostic indicator of the OS of patients with GC. Mechanistically, ZFP64 overexpression induced a GC cell stem-like phenotype and established an immunosuppressive microenvironment by inducing GAL-1 transcription. Moreover, MAPK and PI3K signaling were responsible for the gain of the stem-like phenotype of GC cells, and individual inhibitors of MAPK and PI3K signaling partially sensitized GC cells to nab*-*paclitaxel therapy both in vitro and in vivo, while OTX008 reversed drug resistance and the immunosuppressive tumor microenvironment induced by elevated ZFP64 expression in vivo. In these contexts, we elucidated a new mechanism of GC chemoresistance and found that a high level of ZFP64 plays a critical role in GC progression and chemotherapy resistance by regulating the stem cell-like phenotype and suppressing pathogenic immune activation, suggesting that the disruption of ZFP64-related signaling may inhibit the development of GC and reverse nab*-*paclitaxel resistance in GC (Fig. 9).

As a transcription factor, ZFP64 modulates Notch signaling and is recruited to the promoters of the Notch target genes Hes1 and Hey1 [11], while ZFP64 has also been shown to increase cytokine expression to subsequently activate Toll-like receptor via the NF-κB pathway [25]. Recently, ZFP64 was identified to be a crucial TF in *MLL*-rearranged leukemia by controlling the expression of the oncogene *MLL*, prompting the formal proposal that ZFP64 was an important TF in cancer progression [12]. In fact, several high-throughput analyses indicated that ZFP64 dysregulation might play a vital role in cancer development; for example, a proteomic analysis revealed the upregulation of ZFP64 in samples of liver metastasis of colorectal carcinoma compared to primary cancer samples, and high expression (more than median) of ZFP64 correlated significantly with vascular invasion and potentially represents a prognostic marker of metastasis and death in patients with hepatocellular carcinoma [26, 27]. Additionally, the promoter region of the ZFP64 gene is frequently altered or methylated in GC [14]. Here, ZFP64 was upregulated in GC samples compared to the corresponding paratumor samples, particularly GC tissues from patients who were resistant to nab-paclitaxel treatment. Using RNA-seq and ChIP-seq analyses, we found that elevated ZFP64 expression conferred a stem cell-like phenotype to tumor cells and promoted immunosuppression in the tumor via the transcriptional activation of GAL-1 in GC. It is important to mention that we noticed LGR6, a stemness-related gene, were also detected by ChIP-seq, while the fluorescence gene reporter assay failed to confirm the direct regulation of LGR6 by ZFP64, confirming ZFP64 regulates cell stemness in an indirectly manner. Moreover, patients with high ZFP64 expression had shorter survival times than patients with low ZFP64 expression, and ZFP64 levels potentially represent an independent prognostic factor for patients with GC. Thus, a high level of ZFP64 is a key promoter of GC progression.

Based on accumulating evidence, GAL-1 is instrumental in promoting the progression of cancer [28]. For example, elevated GAL-1 expression has been shown to be involved in neoplastic transformation, cell survival, tumor angiogenesis and metastasis [29]. Importantly, a high level of GAL-1 modulates the immune and inflammatory responses and might play a key role in helping tumor cells escape immune surveillance [24]. For example, GAL-1 expression is correlated with aggressive phenotypes in many types of tumors, including GC, and Gal-1 modulates T cell-mediated immune responses through its pro-apoptotic effect on pro-inflammatory Th subsets and induction of IL-10 synthesis and Th2 cytokine production [30]. Moreover, the abrogation of tumor-derived GAL-1 favors tumor rejection by increasing antitumor T cell proliferation and IFN-γ levels [30]. Mechanistically, dysregulation of GAL-1 expression induced the activation of the Ras/Raf/extracellular signal-regulated kinase (ERK1/2) and PI3K-AKT pathways in cancer cells [31]. Consequently, GAL-1 has become an attractive target for cancer therapeutics [29]. However, researchers have not yet determined which TF induces GAL-1 expression. Here, we first revealed that ZFP64 induces the transcription of GAL-1 in GC cells. Importantly, a high level of GAL-1 enhanced the CSC properties of GC cells via the MAPK and PI3K/AKT pathways, consistent with a previous report. Furthermore, high GAL-1 expression induced the apoptosis of CD8^+^ T cells, which was further confirmed by the GC TMA. Importantly, the administration of OTX008, a GAL-1 inhibitor, exerted a synergistic effect with a nab-paclitaxel treatment on nude mice, particularly humanized NSG mice, indicating that the inhibition of GAL-1 reversed the nab-paclitaxel resistance of GC. Thus, a subgroup of patients with GC presenting high ZFP64 expression in tumors and resistance to nab-paclitaxel treatment might benefit from treatment with the combination of nab-paclitaxel plus OTX008.

## Conclusion

In the present study, we demonstrated ZFP64 promoted GC by endowing GC cells with properties of CSCs and impairing anticancer immune responses via the transcriptional activation of GAL-1. Furthermore, a pharmacological inhibitor of GAL-1, OTX008, may enhance the efficacy of nab-paclitaxel therapy in a subgroup of patients with GC. Taken together, our findings provide a promising approach to overcome chemoresistance and improve GC treatment.

## Supplementary Information

**Additional file 1: Figure S1**. (A) qRT-PCR analysis was used to verify the results of RNA-seq by investigating the expression of 15 genes (random selected) in gastric cancer and paratumor tissues. (B) Correlation of qRT-PCR detection and the indicated genes from RNA-Seq in gastric cancer. (C) ZFP64 mRNA expression in 408 gastric cancer specimens and 211 normal specimens from TCGA database. **Figure S2**. (A) The indicated GC cell lines were treated with nab-paclitaxel for 72 h, and the dose-response curves were shown according to different concentration. (B) Apoptosis rate of different groups. (C) qRT-PCR analysis of indicated genes in ZFP64-overexpressive HGC-27 cells and control cells. (D) Correlation of qRT-PCR detection and the indicated genes from RNA-Seq in ZFP64-overexpressive HGC-27 cells. **Figure S3**. (A-D) ZFP64 and vector-transfected HGC27 and MGC-803 cells were treated with 5-Fu, Cisplatin, Oxaliplatin or Irinotecan for 72 h. Cell viability was quantified by CCK8 assay and IC50 values were calculated. Data represent means ± SEM. * *P* < 0.05, ** *P* < 0.01, *** *P* < 0.005. **Figure S4**. (A-B) Pie graphs showing the distribution of chromatin occupancy peak location. (C) Luciferase reporter assay revealed the luciferase activity of wild and mutant GAL1 promoter by upregulation of ZFP64. Data represent means ± SEM. **** *P* < 0.001, ns, nonsignificant. **Figure S5**. (A) ZFP64 mRNA expression in subcutaneous xenograft tumors. (B) Representative images of orthotopic tumor model. Data represent means ± SEM. *** *P* < 0.005. **Table S1**. List of primary antibodies used in the study. **Table S2**. Primers of genes used in the study. **Table S3**. The correlation between ZFP64 and clinicopathologic features in 420 GC Patients.

## Data Availability

All data generated or analyzed during this study are included in this published article and its supplementary information files.

## Abbreviations

GC: Gastric cancer
nab*-*paclitaxel: nanoparticle albumin-bound paclitaxel
ZFP64: Zinc Finger Protein 64
GAL-1: Galectin-1
PDX: Patient-derived subcutaneous xenografts
CSCs: Cancer stem cells
TMA: Tissue microarray
TNM: Tumor node metastasis
qRT-PCR: Quantitative real-time polymerase chain reaction
OS: Overall survival
EGF: Epidermal growth factor
FGF2: Fibroblast growth factor-2
GSEA: Gene Set Enrichment Analysis
TSS: Transcription start sites
IHC: Immunohistochemistry
NGS: Next-generation sequencing
TF: Transcriptional factor
ChIP: Chromatin immunoprecipitation
