# Supplementary material for: Targeting ZFP64/GAL-1 axis promotes therapeutic effect of nab-paclitaxel and reverses immunosuppressive microenvironment in gastric cancer
Source: J Exp Clin Cancer Res. 2022 Jan 7;41:14. doi: 10.1186/s13046-021-02224-x (PMC8740411; doi:10.1186/s13046-021-02224-x)
Supplement: Supplementary file 1 — Additional file 1: Figure S1. (A) qRT-PCR analysis was used to verify the results of RNA-seq by investigating the expression of 15 genes (random selected) in gastric cancer and paratumor tissues. (B) Correlation of qRT-PCR detection and the indicated genes from RNA-Seq in gastric cancer. (C) ZFP64 mRNA expression in 408 gastric cancer specimens and 211 normal specimens from TCGA database. Figure S2. (A) The indicated GC cell lines were treated with nab-paclitaxel for 72 h, and the dose-response curves were shown according to different concentration. (B) Apoptosis rate of different groups. (C) qRT-PCR analysis of indicated genes in ZFP64-overexpressive HGC-27 cells and control cells. (D) Correlation of qRT-PCR detection and the indicated genes from RNA-Seq in ZFP64-overexpressive HGC-27 cells. Figure S3. (A-D) ZFP64 and vector-transfected HGC27 and MGC-803 cells were treated with 5-Fu, Cisplatin, Oxaliplatin or Irinotecan for 72 h. Cell viability was quantified by CCK8 assay and IC50 values were calculated. Data represent means ± SEM. * P < 0.05, ** P < 0.01, *** P < 0.005. Figure S4. (A-B) Pie graphs showing the distribution of chromatin occupancy peak location. (C) Luciferase reporter assay revealed the luciferase activity of wild and mutant GAL1 promoter by upregulation of ZFP64. Data represent means ± SEM. **** P < 0.001, ns, nonsignificant. Figure S5. (A) ZFP64 mRNA expression in subcutaneous xenograft tumors. (B) Representative images of orthotopic tumor model. Data represent means ± SEM. *** P < 0.005. Table S1. List of primary antibodies used in the study. Table S2. Primers of genes used in the study. Table S3. The correlation between ZFP64 and clinicopathologic features in 420 GC Patients. [file 13046_2021_2224_MOESM1_ESM.pdf]

Supplementary Figure legends

Figure S1 (A) qRT-PCR analysis was used to verify the results of RNA-seq by investigating the expression of 15 genes (random selected) in gastric cancer and paratumor tissues. **(B)** Correlation of qRT-PCR detection and the indicated genes from RNA-Seq in gastric cancer. **(C)** ZFP64 mRNA expression in 408 gastric cancer specimens and 211 normal specimens from TCGA database.

Figure S2 (A) The indicated GC cell lines were treated with nab-paclitaxel for 72 hours, and the dose-response curves were shown according to different concentration. **(B)** Apoptosis rate of different groups. **(C)** qRT-PCR analysis of indicated genes in ZFP64-overexpressive HGC-27 cells and control cells. (**D**) Correlation of qRT-PCR detection and the indicated genes from RNA-Seq in ZFP64-overexpressive HGC-27 cells.

Figure S3 (A-D) ZFP64 and vector-transfected HGC27 and MGC-803 cells were treated with 5-Fu, Cisplatin, Oxaliplatin or Irinotecan for 72h. Cell viability was quantified by CCK8 assay and IC50 values were calculated. Data represent means ± SEM. * $P < 0.05$, ** $P < 0.01$, *** $P < 0.005$.

Figure S4 (A-B) Pie graphs showing the distribution of chromatin occupancy peak location. **(C)** Luciferase reporter assay revealed the luciferase activity of wild and mutant GAL1 promoter by up-regulation of ZFP64. Data represent means ± SEM. **** $P < 0.001$, ns, nonsignificant.

Figure S5 (A) ZFP64 mRNA expression in subcutaneous xenograft tumors. **(B)** Representative images of orthotopic tumor model. Data represent means ± SEM. *** $P < 0.005$

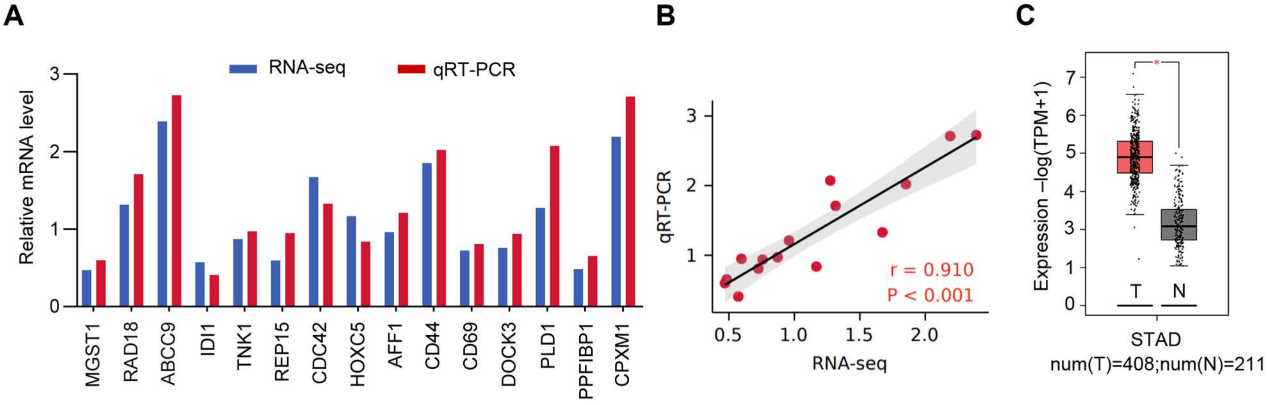

A
RNA-seq
qRT-PCR
Relative mRNA level
3
2
1
0
MGST1
RAD18
ABCC9
IDI1
TNK1
REP15
CDC42
HOXC5
AFF1
CD44
CD69
DOCK3
PLD1
PPFIBP1
CPXM1
B
qRT-PCR
3
2
1
r = 0.910
P < 0.001
0.5
1.0
1.5
2.0
RNA-seq
C
Expression −log(TPM+1)
7
6
5
4
3
1
0
*
T
N
STAD
num(T)=408;num(N)=211

Figure S1

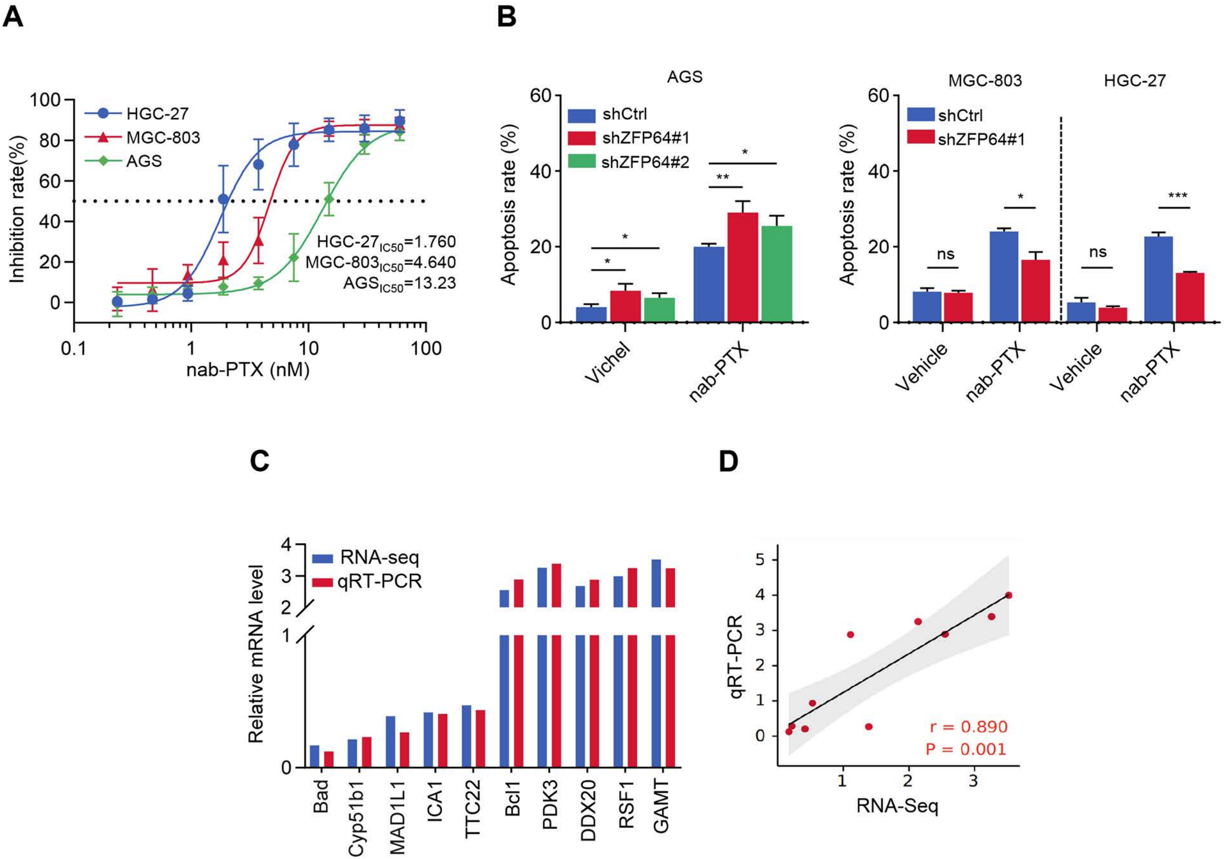
A
B
C
D
Inhibition rate(%)
nab-PTX (nM)
HGC-27
MGC-803
AGS
HGC-27IC50=1.760
MGC-803IC50=4.640
AGSIC50=13.23
AGS
MGC-803
HGC-27
Apoptosis rate (%)
shCtrl
shZFP64#1
shZFP64#2
Vichel
nab-PTX
Vehicle
ns
Relative mRNA level
RNA-seq
qRT-PCR
Bad
Cyp51b1
MAD1L1
ICA1
TTC22
Bcl1
PDK3
DDX20
RSF1
GAMT
qRT-PCR
RNA-Seq
r = 0.890
P = 0.001

Figure S2

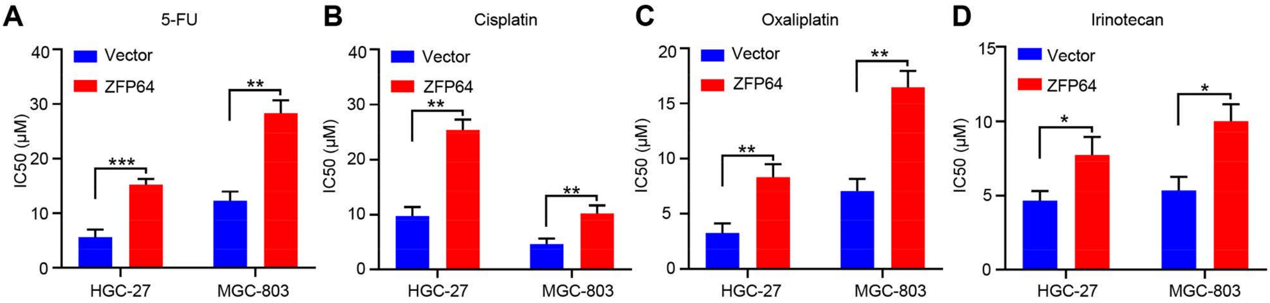
A
5-FU
B
Cisplatin
C
Oxaliplatin
D
Irinotecan
Vector
ZFP64
IC50 (μM)
HGC-27
MGC-803
***
**
*

Figure S3

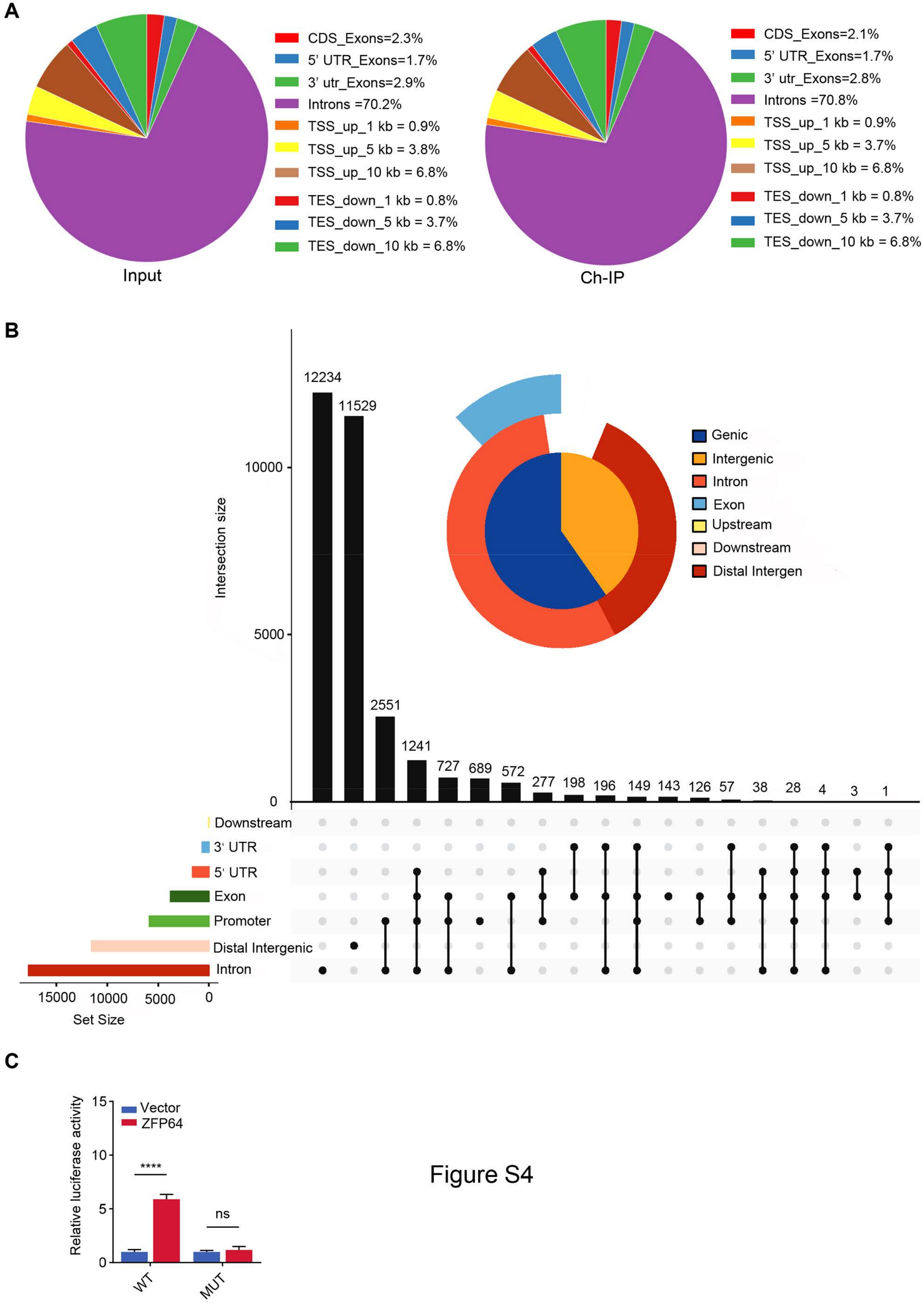
A
CDS_Exons=2.3%
5' UTR_Exons=1.7%
3' utr_Exons=2.9%
Introns =70.2%
TSS_up_1 kb = 0.9%
TSS_up_5 kb = 3.8%
TSS_up_10 kb = 6.8%
TES_down_1 kb = 0.8%
TES_down_5 kb = 3.7%
TES_down_10 kb = 6.8%
Input
CDS_Exons=2.1%
5' UTR_Exons=1.7%
3' utr_Exons=2.8%
Introns =70.8%
TSS_up_1 kb = 0.9%
TSS_up_5 kb = 3.7%
TSS_up_10 kb = 6.8%
TES_down_1 kb = 0.8%
TES_down_5 kb = 3.7%
TES_down_10 kb = 6.8%
Ch-IP
B
Intersection size
0
5000
10000
12234
11529
2551
1241
727
689
572
277
198
196
149
143
126
57
38
28
4
3
1
Genic
Intergenic
Intron
Exon
Upstream
Downstream
Distal Intergen
Downstream
3' UTR
5' UTR
Exon
Promoter
Distal Intergenic
Intron
15000
10000
5000
0
Set Size
C
Relative luciferase activity
0
5
10
15
Vector
ZFP64
****
ns
WT
MUT

Figure S4

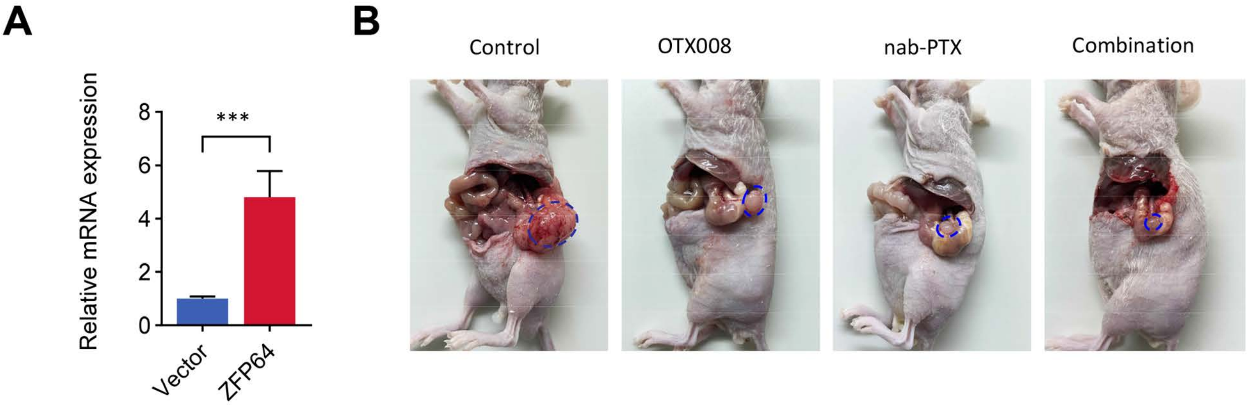
A
B
Control
OTX008
nab-PTX
Combination
Relative mRNA expression
8
6
4
2
0
***
Vector
ZFP64

Figure S5

Table S1. List of primary antibodies used in the study.

Antibody	Applications	Company
ZFP64	IP, WB	Abcam (ab117787)
ZFP64	IF, IHC	Abcam (ab204610)
Galectin 1	F, IHC, WB	Abcam (ab138513)
Vimentin	F, IF, IHC, WB	Abcam (ab8978)
E-Cadherin	F, IHC, WB	Abcam (ab231303)
ERK1/2	WB, IHC, IF, F	CST (4696)
p-ERK1/2	WB, IF, IHC, IP	CST (4370)
ALDH1A1	WB, IP, IHC, IF, ELISA	Proteintech (15910-1-AP)
LGR6	WB, IHC, F, ELISA	Proteintech (17658-1-AP)
AKT	WB, IP, IHC, IF, F, ChIP	CST (4685)
p-AKT	WB, IP, IF, IHC	CST (4060)
CD8	WB, IHC, IF	Abcam (ab93278)
CD44	WB, IP, IF, F	CST (5640)
CD44	F	eBioscience (17-0441-83)
IgG	WB, IHC, IP, CHIP, F	Abcam (ab172730)
GAPDH	WB, IP, IF	Proteintech (60004-1-Ig)

Abbreviations: WB, western blot; IHC, immunohistochemistry; IF, immunofluorescence; IP, immunoprecipitation; ELISA, enzyme-linked immunosorbent assay; FCA, flow cytometric analysis; CHIP, Chromatin Immunoprecipitation.

Table S2. Primers of genes used in the study.

Genes	Forward Sequence (5' -> 3')	Reverse Sequence (5' -> 3')
ZFP64	AGGTTGCCAATTCAAGACTGC	CACCGCATGTGAGTTTTCAGC
GAL-1	CTGTGCCTGCACTTCAACC	CATCTGGCAGCTTGACGGT
MGST1	ATTGGCCTCCTGTATTCCTTGA	GTGCTCCGACAAATAGTCTGAAG
RAD18	GAGCCGGATCTGAAAAATAACCG	GCTGGTGACTCTAAAGCAAACTG
ABCC9	TTACATTCGCTCTCCTGTTTGTG	GGTGGAGGTGCCTTGATTCC
IDI1	AACACTAACCACCTCGACAAGC	AGACACTAAAAGCTCGATGCAA
TNK1	TGAAAAGGCTACGTTCTGGGC	ACCCTCTGGGATCAGACACTT
REP15	AAGAAAAGGGAGTTGATGAGTGG	TTCTGGATTGGAAAGGTCACAAG
HOXC5	CTAAGAGCAGTGGGGAGATCA	GTCATCCACGGGTAAATCTGTG
CDC42	GTCATCCACGGGTAAATCTGTG	CCATCGGAATATGTACCGACTG
CD69	ATTGTCCAGGCCAATACACATT	CCTCTCTACCTGCGTATCGTTTT
AFF1	GCCAAGTCTCCATGCCAAAAG	CTTGTGATGACTAGAGCCGAAC
CD44	CTGCCGCTTTGCAGGTGTA	CATTGTGGGCAAGGTGCTATT
DOCK3	ACAAGAATGGGCAAGTTTGTGG	TCATCACATGGCGTAGTTTGTAG
PLD1	CCCAGCGATCCCAAGATACAA	GACAGCCGGAGAGATACGTCT
PPFIBP1	ACAAGTGTTCCCGAAGAGTTCC	CACAGTTGGGTATCAACAGTGG
CPXM1	GGCGATCTATATGATGGAGCCT	CCTGTGTGATAACACCCGAGAA
Bad	CCCAGAGTTTGAGCCGAGTG	CCCATCCCTTCGTCGTCCT
CYP51A1	GAAACGCAGACAGTCTCAAGA	ACGCCCATCCTTGTATGTAGC

ICA1	CTTCGATCCCAAGGTTTCCAA	TCGACACAAAGGATTTCGTAAGG
TTC22	GCAGTTGAACTTCGAGCCG	TCCTCCAGGTAGAATGCGAAA
Bcl1	GCTGCGAAGTGGAAACCATC	CCTCCTTCTGCACACATTTGAA
PDK3	CGCTCTCCATCAAACAATTCCT	CCACTGAAGGGCGGTTAAGTA
DDX20	GCTGCGGGCTCGATTTAATTG	GTCCAAAGCTATGGTGGAGAAC
RSF1	GGATGCCGATACTATGCGTCT	GCCAACTCGTTTCGATTTCTGA
GAMT	CGCCCATTGATGAGCATTGG	GGCCTTTCAAGGGGATGACC
MAD1L1	TGGACTGGATATTTCTACCTCGG	CCTCACGCTCGTAGTTCCTG
LGR6	TGGGGAACCCTCTGCTACAG	CAGGTACTGGAATGCCGATCT
ALDH1A1	GCACGCCAGACTTACCTGTC	CCTCCTCAGTTGCAGGATTAAAG
GAPDH	CTGGGCTACACTGAGCACC	AAGTGGTCGTTGAGGGCAATG

Table S3. The correlation between ZFP64 and clinicopathologic features in 420 GC Patients.

Variable	Number of Patients		p-value*
	ZFP64^{low} (N=240)	ZFP64^{high} (N=180)	
**Age**			
<55	89	58	0.352
≥55	151	122	
**Gender**			
Female	112	67	
Male	128	113	0.058
**Lauren's type**			
Intestinal type	124	99	0.553
Non-intestinal type	116	81	
**Lymph node metastasis**			
Negative	87	24	P<0.001
Positive	153	156	
**pTNM stage**			
I-III	122	45	P<0.001
IV	188	135	
**Cancer thrombus in vessel**			
Negative	163	88	P<0.001
Positive	77	92	

Note: A Chi-square test was used for comparing groups between low and high ZFP64 expression.

*$P<0.05$ was regarded as statistically significant.
